# Containing Ebola at the Source with Ring Vaccination

**DOI:** 10.1371/journal.pntd.0005093

**Published:** 2016-11-02

**Authors:** Stefano Merler, Marco Ajelli, Laura Fumanelli, Stefano Parlamento, Ana Pastore y Piontti, Natalie E. Dean, Giovanni Putoto, Dante Carraro, Ira M. Longini, M. Elizabeth Halloran, Alessandro Vespignani

**Affiliations:** 1 Bruno Kessler Foundation, Trento, Italy; 2 Laboratory for the Modeling of Biological and Socio-technical Systems, Northeastern University, Boston, Massachusetts, United States of America; 3 Department of Biostatistics, College of Public Health and Health Professions, University of Florida, Gainesville, Florida, United States of America; 4 Doctors with Africa-CUAMM, Padua, Italy; 5 Vaccine and Infectious Disease Division, Fred Hutchinson Cancer Research Center, Seattle, Washington, United States of America; 6 Department of Biostatistics, University of Washington, Seattle, Washington, United States of America; 7 Institute for Quantitative Social Sciences at Harvard University, Cambridge, Massachusetts, United States of America; 8 Institute for Scientific Interchange Foundation, Turin, Italy; London School of Hygiene and Tropical Medicine, UNITED KINGDOM

## Abstract

Interim results from the Guinea Ebola ring vaccination trial suggest high efficacy of the rVSV-ZEBOV vaccine. These findings open the door to the use of ring vaccination strategies in which the contacts and contacts of contacts of each index case are promptly vaccinated to contain future Ebola virus disease outbreaks. To provide a numerical estimate of the effectiveness of ring vaccination strategies we introduce a spatially explicit agent-based model to simulate Ebola outbreaks in the Pujehun district, Sierra Leone, structurally similar to previous modelling approaches. We find that ring vaccination can successfully contain an outbreak for values of the effective reproduction number up to 1.6. Through an extensive sensitivity analysis of parameters characterising the readiness and capacity of the health care system, we identify interventions that, alongside ring vaccination, could increase the likelihood of containment. In particular, shortening the time from symptoms onset to hospitalisation to 2–3 days on average through improved contact tracing procedures, adding a 2km spatial component to the vaccination ring, and decreasing human mobility by quarantining affected areas might contribute increase our ability to contain outbreaks with effective reproduction number up to 2.6. These results have implications for future control of Ebola and other emerging infectious disease threats.

## Author Summary

When the 2014–15 Ebola outbreak in West Africa began, no licensed vaccines for the disease were available. The rVSV-ZEBOV vaccine was developed during the course of the epidemic and underwent a clinical trial demonstrating 100% efficacy when vaccinating contacts and contacts of contacts of confirmed Ebola cases (an approach called ring vaccination). However, the trial did not provide any understanding on whether this vaccination strategy can be effective in containing future Ebola virus disease outbreaks. Through a modelling study on a region of Sierra Leone, we provide numerical estimates for the effectiveness of ring vaccination: we show that outbreaks with moderate transmission potential, with no more than 1.6 secondary cases generated by an index case on average, can be successfully contained; more extensive vaccination(e.g., including spatial rings around index cases) and reinforcement of the healthcare system would increase the likelihood of containment even if the virus were more transmissible than in the past. Our results provide implications for control plans of possible future Ebola outbreaks.

## Introduction

The 2014–15 Ebola virus disease (EVD) epidemic in West Africa was the largest Ebola outbreak ever documented with a total of 28,646 cases and 11,323 deaths reported as of March 30, 2016 [1, 2]. At the start of the epidemic, no licensed vaccines were available for Ebola.

In July 2015, Henao-Restrepo and colleagues [3, 4] demonstrated 100% (95% confidence interval (CI): 74.7–100.0) efficacy of the rVSV-ZEBOV vaccine against EVD using a ring vaccination cluster randomised trial in Guinea. Rings, *i*.*e*., clusters, were defined as the contacts and contacts of contacts of confirmed EVD cases. Ring vaccination strategies were instrumental in the elimination of local outbreaks of smallpox during the eradication phase [5]. Several modelling studies evaluating targeted smallpox vaccination strategies have been performed (see for instance [6, 7]). Modelling results highlighted the time from symptom onset to case isolation and the fraction of contacts identified by contact tracing as the most important factors determining the success of ring vaccination strategies. Questions remain about whether a ring vaccination strategy can be effective in containing EVD outbreaks, such as the EVD flare-up observed in West Africa in 2016 [2].

We use a spatially explicit agent-based model of EVD transmission based on a detailed synthetic population of Sierra Leone (see Methods and SI) [8, 9]. The model is calibrated to reproduce the most important features of the EVD outbreak in Pujehun district, that can be considered typical in terms of both transmissibility and key time periods. Specifically, the observed basic reproduction number was *R*_0_ = 1.63, in the range of values estimated for West Africa of 1.6–2.2 [8, 10–13]. Furthermore the average generation time of 13.7 days, and the average incubation time of 9.7 days are similar to the WHO estimates of 15.3 days and 11.4 days [10], respectively.

Here we examine the effectiveness of ring vaccination with the rVSV vaccine in containing the spread of EVD. We focus on possible future EVD outbreaks reemerging in West Africa. We assume, however, that the existing infrastructure for managing the 2014–15 EVD epidemic has at least partly dissolved. Results apply also to the emergence of EVD in different African countries where this infrastructure is not present at all.

In this context, we assume that ring vaccination will be the primary containment strategy. Specifically, we consider the following paradigm for containing future EVD outbreaks: i) isolation of index cases and identification of contacts and contacts of contacts of index cases and ii) vaccination of contacts and contacts of contacts. Additional non-pharmacological interventions, early isolation of secondary cases through contact tracing, will eventually be implemented on top of vaccination.

We focus on ring vaccination’s ability to contain an early outbreak by reporting the epidemic prevention potential (EPP [17–19] see Methods). The EPP reflects the probability of an intervention containing an outbreak. We consider different levels of uncertainty and different values of the effective reproduction number *R_e_*, the average number of secondary cases generated by a primary infector. This analysis allows us to identify the main determinants of the probability of outbreak containment, and expands upon previous modeling work [20, 21] as we consider the important impact of varying the ring definition (contacts, contacts of contacts, and spatial components), as well as the performance of ring vaccination assuming different levels of *R_e_* and contact tracing.

## Methods

### Transmission model

Our model (see the S1 File for details) considers the Pujehun district of Sierra Leone. The model population of 375,000 individuals was assigned to one central town (population 30,000) and neighbouring villages, reflecting district-level data from the Demographic and Health Survey [22]. Individuals are assigned to specific households, and households are linked to create “extended households” as are typical in rural Africa. The model is an extension of a model used to simulate interventions during the 2014 outbreak [9]. We used microsimulations to explicitly model Ebola transmission within households and extended households (35.9% and 38.5% of transmission in Pujehun district, respectively; 74.4% of transmission combined) and in the general community, including non-household and health care-related contacts (25.6% of transmission) [9]. Ebola transmission occurring during burial ceremonies is captured within these estimates. The extended household defines the set of contacts and contacts of contacts that could be identified through contact tracing. As a baseline we assume that all contacts of the extended household (74.4% of transmission combined) can be identified through contact tracing. We also consider a more optimistic scenario where we assume that 90% of contacts can be identified through contact tracing. This assumption stems from the observation that most of non-family transmission events in Pujehun district were observed among friends [9] and thus they could be potentially traceable, at least to some extent. The relative proportions of transmissions are consistent with findings from other West African regions [8, 15, 16]. The model allows for heterogeneity in transmission [9, 14] and for variation in age-specific risk of infection [9].

### Effective reproduction number

Because we used a heterogeneous transmission model and included the contact structure of the population, the distribution of secondary cases per index case has a long tail with non-negligible probabilities for 10 or more secondary cases. In Pujehun the distribution of secondary cases was overdispersed, resembling a negative binomial with dispersion parameter *k* = 0.45, comparable to *k* = 0.18 observed in Conakry, Guinea [14]. Furthermore, most of the transmission events occurred among close contacts, with 74.3% of cases exposed in family or extended family, similar to the 72% estimated for Conakry, Guinea [15] and to the 71.4% observed in Montserrado, Liberia [16]. In our model we considered *R_e_* values for EVD from 1.4 to 2.6, which extends beyond the plausible range of values estimated in West Africa (1.6 to 2.2) [8, 10–13]. In fact, the transmission potential for future EVD outbreaks, possibly originating in different regions of Africa, could be different from that observed in West Africa. For instance, it could be either decreased or increased by human behaviour, by the ability of health care workers to limit transmission in health care facilities, and by burial procedures. Key model parameters and their assumed values are summarised in Table 1.

**Table 1:** Model parameter values.

Parameter	Baseline	Reference	Explored range
Incubation period	10 days	[9]	–
Symptoms onset to hospitalization	4 days	[9]	1–6 days
Symptoms onset to death/recovery for unhospitalized cases	6.5 days	[9]	–
Hospitalization to discharge	9 days	[9]	–
Hospitalization to death	2 days	[9]	–
Case fatality ratio	85.7%	[9]	–
Ring enrolment to immunity^#^	6 days	[4]	4–12 days
Vaccination policy*	C&CC	[4]	C, C&CC, S, C+S C&CC+S, M
Radius of the spatial ring	0 km	[4]	0–20 km
Vaccine coverage of eligible population	65%	[4]	50–95%
Number of EVD cases to detect the outbreak	5	Arbitrary	1–20
Number of Ebola beds	20	[9]	1–40
Percentage of isolated EVD cases	88.8%	[9]	30–88.8%
Spatial transmission kernel (parameter *b*)^$^	2.25	[23]	1–4
Heterogeneous transmission (parameter *ρ*)^+^	0.45	[9]	0.2-*∞*
Traceable contacts (among all contacts)	74.4%	[9]	74.4–90%

* C: contacts of index cases; CC: contacts of contacts; S: spatial ring; M: mass vaccination

^#^ 2 days to define the ring, obtain consent, and administer vaccine, followed by 4 days to develop protective immunity [4].

^$^ Force of infection in the general community decreases with distance (*d*) proportionally to the kernel 1/(1 + *d^b^*).

^+^ Individual infectiousness is sampled from a Gamma distribution with mean 1 and shape *ρ*. Thus, the number of secondary cases has a negative binomial distribution with dispersion *ρ*.

### Epidemic prevention potential

We measure the impact of ring vaccination on simulated outbreaks in terms of EPP [17], defined as EPP = 1 − *p_I_* /*p_NI_*, where *p_I_* and *p_NI_* are the probabilities of an uncontained outbreak when an intervention is used (e.g. ring vaccination) or no intervention is used, respectively. Here an outbreak is considered uncontained if the number of cases exceeds 300, though the results are consistent if this threshold is varied by up to 30%. Because the probability of an uncontained outbreak increases with *R_e_*, the EPP measures the impact of an intervention in preventing an uncontained outbreak, standardising by the background probability of an uncontained outbreak. An EPP of 1 indicates that an intervention always contains an otherwise uncontained outbreak, while an EPP of 0 indicates that the intervention never contains an outbreak that would have been uncontained. We provide estimates of the number of vaccine doses required to implement ring vaccination. In particular, we report the number of vaccine doses required for successful containment only. Indeed, for an uncontained outbreak, it is likely that the epidemic would invade other districts and neighbouring countries. Instead of local containment, intervention measures would likely focus on outbreak mitigation, and vaccination strategies would be integrated with other intervention options.

## Results

**Fig 1:**
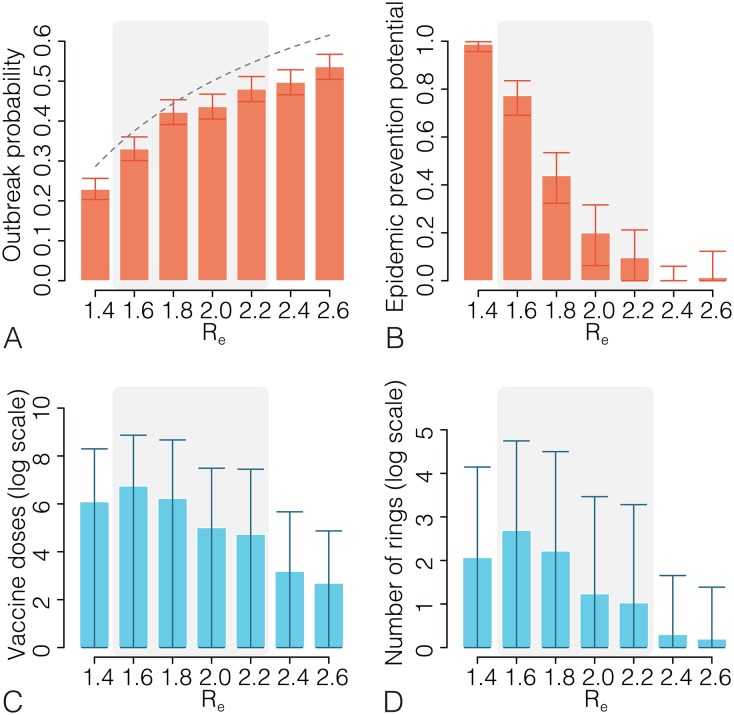
Ring vaccination with baseline parameters. **A** Probability (with 95%CI; exact binomial test) of an uncontained outbreak (more than 300 cases) after introducing one infected case into a fully susceptible population for a range of *R_e_* values. The dashed line represents the theoretical value 1 − 1/*R_e_* under a homogeneous mixing assumption. **B** Estimated epidemic prevention potential and 95%CI for a range of *R_e_* values. The shaded grey area denotes the most plausible *R_e_* values for the 2014–15 West African epidemic. **C** Mean number of vaccine doses and 95%CIs for containing an outbreak for a range of *R_e_* values. **D** As C but for the number of rings defined. Each estimate is based on 1,000 simulated outbreaks.

By assuming baseline values of model parameters (see Table 1), the probability of an uncontained outbreak in the no-intervention scenario is shown in Fig 1A. The no-intervention scenario for a randomly mixing population considers a situation with little or no readiness of the health care systems—a reasonable assumption for the very early phase of the outbreak—and with an effective reproductive number that accounts for the baseline practices for transmission control. The outbreak probability increases from about 23% for *R_e_* = 1.4 to 54% for *R_e_* = 2.6. Outbreak probabilities are lower than what would be theoretically predicted by *p* = 1 − 1/*R_e_*. This is expected due to the heterogeneity in transmission [24]. The impact of ring vaccination is reported in Fig 1B. The EPP of ring vaccination is near 1 when *R_e_* ≤ 1.4. The EPP of ring vaccination declines as *R_e_* increases, decreasing to approximately 0.40 when *R_e_* = 1.8, and to nearly 0 when *R_e_* ≥ 2.4. The probability of an uncontained outbreak by assuming baseline ring vaccination increases from about 0.3% for *R_e_* = 1.4 to 53% for *R_e_* = 2.6. When *R_e_* = 1.6, our model returns an estimated vaccine effectiveness (see S1 File) consistent with the 75.1% effectiveness observed in the Guinea ring vaccination trial [4] (see S1 File). On average, 14 rings (max 169) and 847 vaccine doses (max 10,659) are required to contain an outbreak when *R_e_* = 1.6 (Fig 1C and 1D). The mean number of cases with baseline ring vaccination ranges from 15.5 (95%CI:1–121) for *R_e_* = 1.4 to 2.0 (95%CI:1–12) for *R_e_* = 2.6: in fact, for small values of *R_e_* it is possible to contain the outbreak with ring vaccination even after a few generations of cases, while for large values of *R_e_* the outbreak either will be contained early or it will be not contained. These results suggest that ring vaccination strategies with baseline intervention parameters can be highly effective and affordable for containing an EVD outbreak when *R_e_* ≤ 1.6.

**Fig 2:**
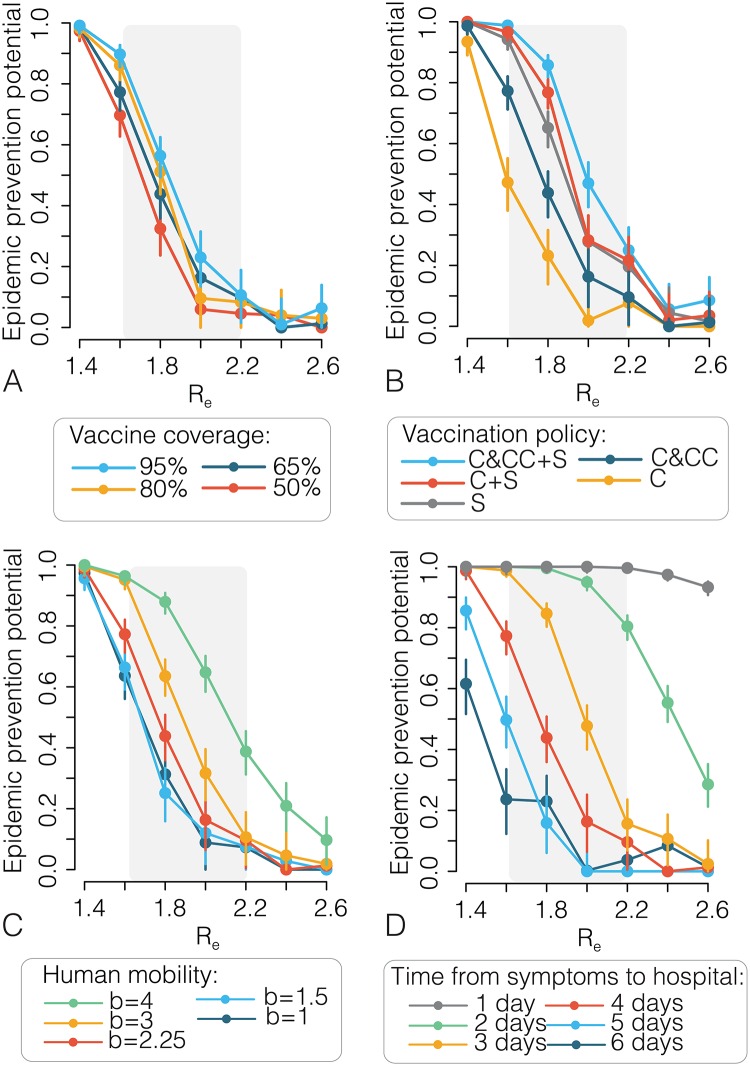
Sensitivity analysis. **A** Estimated epidemic prevention potential (points) and 95%CI (vertical lines) as a function of *R_e_* and by varying the vaccine coverage. Baseline coverage: 65% [4]. The shaded grey area represents the range of most plausible values of *R_e_* for the 2014–15 epidemic in West Africa. **B** As in **A** but by varying the eligible population. Symbols: C indicates contacts of index cases; CC indicates contacts of contacts; S indicates geographical rings (ring radius: 2 km). Baseline value: C&CC [4]. **C** As in **A** but by varying the parameter regulating human mobility. Spatial transmission is proportional to a power law kernel 1/(1 + d^b^) where *d* is the geographical distance and *b* regulates the decrease of transmission with distance. Baseline value: *b* = 2.25, resulting in an average distance of 7.7 km [23]. Other scenarios assume different values of *b*, corresponding to an average distance ranging from 1.5 to 25 km. In these scenarios, probabilities of outbreaks in the absence of interventions were recomputed for each value of the parameter *b*. **D** As in **A** but by varying the time from symptoms onset to hospitalisation. Baseline value: 4 days [9]. Each estimate is based on 1,000 simulated outbreaks.

Results of an extensive sensitivity analysis on all parameters regulating EVD transmission and interventions are discussed in the S1 File. Briefly, results show that achieving high vaccine coverage within the ring (*≥* 80%), adding a spatial component to the ring definition (2 km), decreasing human mobility, and decreasing the time from symptom onset to isolation to 2–3 days (e.g. through contact tracing) can drastically increase the EPP (Fig 2).

**Fig 3:**
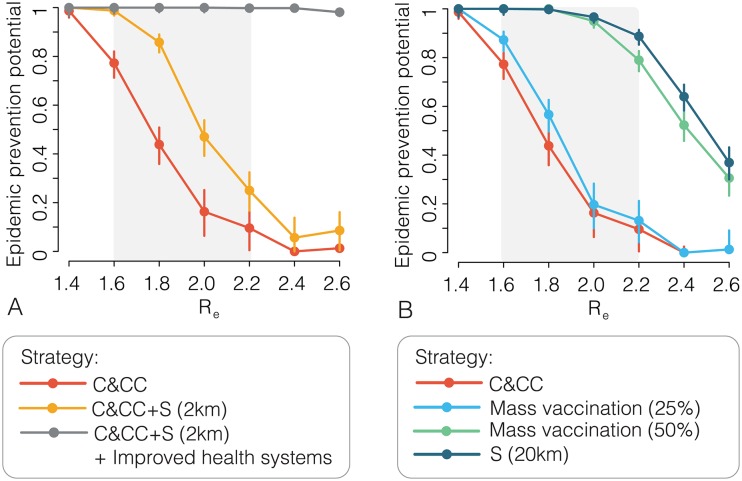
**A** Estimated epidemic prevention potential and 95%CI for a range of *R_e_* values using three ring-defining strategies: contacts and contacts of contacts (C&CC) of index cases; C&CC with a spatial ring of radius 2km around index cases (C&CC+S); C&CC+S (2km) plus “improved health systems”, which includes reducing time to isolation of cases (2–3 days from onset to hospitalisation), increasing ring vaccination coverage to 90% of eligible individuals, and reducing public access to infected areas. The shaded grey area denotes the most plausible *R_e_* values for the 2014–15 epidemic in West Africa. **B** As **A** but considering mass vaccination (coverage 25% and 50%) and a spatial ring (S) of 20km around index cases. Each estimate is based on 1,000 simulated outbreaks.

For values of *R_e_* > 1.6, outbreak containment will likely require additional interventions or higher vaccine coverage and earlier outbreak detection. One option is to add a spatial (S) component to the ring vaccination strategy, vaccinating all eligible individuals within a fixed radius around the index case, (C&CC+S) with 65% coverage. Outbreak containment can be further improved if other interventions are simultaneously implemented, including reducing time to isolation of cases (2–3 days from onset to hospitalisation), increasing ring vaccination coverage to 90% of eligible individuals, and reducing public access to infected areas. We refer to this potentially feasible combination as “improved health systems” and estimate its added impact in Fig 3A. When these interventions are added, the EPP is nearly 1 for all values of *R_e_*. The combinations of interventions discussed above all seem implementable, at least to some extent, considering that i) vaccine safety and efficacy have been demonstrated in children; ii) the average time spent in the community by EVD cases was reduced to 2 days in the last part of the 2014–15 EVD epidemic in West Africa; iii) a spatial ring of about 2 km requires targeting a very limited number of villages; iv) reducing human mobility can be achieved through roadblocks (e.g., through checkpoints with medical screening, as done during the 2014–15 EVD epidemic in West Africa or defining quarantine areas.

We compare ring vaccination with preemptive and reactive mass vaccination strategies in Fig 3B as these strategies have been considered as alternatives for containing EVD [21]. We consider preemptive mass vaccination covering 25% and 50% of all eligible individuals in the region (corresponding to 93,750 and 187,500 vaccine doses, respectively). We model reactive vaccination as a large spatial (S) ring covering 65% of eligible individuals in an area 20km around the index case. Our results suggest that reactive mass vaccination may be more effective than preemptively mass vaccinating 25–50% of the eligible population, and requires significantly fewer vaccine doses, especially when *R_e_* < 1.8. For instance, if *R_e_* = 1.6, an average of 43,027 vaccine doses overall (upper 95%CL: 188,772) are required to contain an outbreak using reactive mass vaccination.

## Discussion

We have shown that ring vaccination of contacts and contacts of contacts using the highly efficacious rVSV-ZEBOV vaccine can effectively contain an EVD outbreak when *R_e_* ≤ 1.6. Containment is likely to occur for values of *R_e_* up to 1.8 if the outbreak is readily detected and the vaccine coverage in the rings is as high as 80%. Adding a 2km spatial component to the ring definition, corresponding to targeting 2.7 villages on average (SD: 1.3), further enhances the effectiveness of ring vaccination, and reinforcement of the healthcare system plus ring vaccination can all but eliminate the probability of an uncontained outbreak. Specifically, shortening the time from symptoms onset to hospitalisation to 2–3 days on average through contact tracing procedures, adding a 2km spatial component to the ring definition, and decreasing human mobility through quarantine of affected areas would result in likelihood of containment close to 1 for values of *R_e_* up to 2.6. Alternatively, for values of *R_e_* larger than 1.8, reactive mass vaccination in a 20km radius around the index case greatly decreases the probability of an uncontained outbreak and reduces the public health burden while requiring a fraction of the doses necessary for preemptive mass vaccination. However, even in the case of larger values of *R_e_* (e.g. *R_e_* = 1.84 as observed in Liberia [8]) it should be considered that most initial transmission in health care settings, that was relevant in Liberia [8], could be avoided by preventive vaccination of health care workers; thus contributing to reduce the initial *R_e_* and to increase the likelihood of containment with ring vaccination.

Moreover, given the experiences of previous Ebola outbreaks where no vaccine was available (e.g., the 2014 outbreak in Nigeria) and of occasional flare-ups in West Africa that have been rapidly brought under control with traditional control strategies [16], it is likely that traditional non-pharmaceutical measures will still be considered, together with ring vaccination, to manage future EVD outbreaks, thus contributing to further reduce *R_e_*. Our model does not consider all these measures explicitly (only hospitalisation of cases is explicitly simulated). While this is a limitation of our study, we accounted for the direct effects of these additional measures. Indeed, the considered reduction of the time from symptom onset to hospitalisation can be interpreted as the result of contact tracing procedures; the considerable reduction of human mobility can be interpreted as the result of quarantine of affected areas. While the model has limitations, including sensitivity to assumptions about hospitalisation rate, number of Ebola beds, and time until the outbreak is first detected, the results are informative.

Our results should also be discussed in the light of previous modeling works. In [20] the authors evaluate the impact of vaccinating only contacts of cases. The study was published before the publication of the original papers describing the rVSV vaccination trial in West Africa [3, 4], where a different definition of ring vaccination, considering also contacts of contacts, has been used. Difference with our results may be traced back to the fact that our analysis evaluates vaccination strategies including contacts and contacts of contacts. In [21], authors observe that ring vaccination might not lead to containment of future EVD outbreaks. However, results depend on the assumption that cases missed by contact tracing transmit the infection much more than cases in known clusters of transmission (7 and 0.66 cases on average respectively). In particular this implies that the basic reproductive number increases proportionally to the percentage of missed cases. The authors themselves recognize this as a study limitation. We integrate into the model information on the percentage of cases generated in family or extended family (72% of transmission in Conakry, Guinea [15], 71.4% in Montserrado, Liberia [16], and 74.3% of transmission in Pujehun district, Sierra Leone [9]) that allows us to set the lower limit of the percentage of traceable cases. Our approach is supported by the analysis conducted in [16] where authors found that 71.4% (15/21) of cases were listed as contacts.

Our results indicate that the efforts using ring vaccination to contain EVD flare-ups in Guinea and Liberia should be successful. Plans are underway to create a mobile stockpile of rVSV-ZEBOV vaccine at the World Health Organisation (WHO) to contain future Ebola outbreaks [25]. The GAVI alliance has pledged funds to buy 300,000 doses of rVSV-ZEBOV vaccine for such a stockpile [26]. Based on the results here, the planned stockpile of 300,000 doses should be sufficient to implement ring vaccination policies for containing a timely detected Ebola outbreak at the source. If the outbreak is left unchecked and eventually invades other regions/countries obviously much more vaccine doses would be needed in the mitigation and eradication effort. Strategies based on spatial vaccination or mass vaccination might require a greater effort, especially in the case of outbreaks in urban settings.

Targeted vaccination interventions, as outlined here, should be adapted for other emerging infectious disease threats, as was done in the past for smallpox [27]. The use of these control strategies, both for assessing effectiveness and actual containment, is part of the WHO plan for dealing with future emerging infectious disease threats [28]. The work presented here should be instrumental in moving this process forward.

## Supporting Information

S1 File Supporting text. Supporting text containing methodological details and additional results.
(PDF)

S2 File Supporting video. Pujehun district, contained outbreak. The movie shows the simulated weekly number of cases of Ebola for the period of one year considering a reproductive number R_0_ = 1.6. On the left we show the evolution of an outbreak where we do not consider any interventions while on the right we consider a ring vaccination intervention (C&CC, baseline parameters, see Fig 2 in the main text) that results in a contained outbreak (56 cumulative cases), as only few villages are reached by the epidemic. Each dot represents a village in the Pujehun district and the colors indicate the number of cases in each one of them. The lower panel shows the weekly number of cases for the total of the region for both scenarios, without interventions and ring vaccination.
(MP4)

S3 File Supporting video. Pujehun district, no contained outbreak. The movie shows the simulated weekly number of cases of Ebola for the period of one year considering a reproductive number R_0_ = 1.6. On the left we show the evolution of an outbreak where we do not consider any interventions while on the right we consider a ring vaccination intervention (C&CC, baseline parameters, see Fig 2 in the main text) that is not able to contain the outbreak (>300 cumulative cases). However, after one year the number of cases observed is significantly lower (345 and 194,599 with and without intervention respectively). Each dot represents a village in the Pujehun district and the colors indicate the number of cases in each one of them. The lower panel shows the weekly number of cases for the total of the region for both scenarios, without interventions and ring vaccination.
(MP4)

## References

[1] Dixon MG, Schafer IJ, et al Ebola viral disease outbreak—West Africa, 2014. MMWR Morb Mortal Wkly Rep. 2014;63(25):548–51. 24964881 PMC5779383

[2] World Health Organization. Ebola situation report—30 March 2016; 2016. http://apps.who.int/ebola/current-situation/ ebola-situation-report-30-march-2016.

[3] Ebola **ç**a Suffit Ring Vaccination Trial Consortium and others. The ring vaccination trial: a novel cluster randomized controlled trial design to evaluate vaccine efficacy and effectiveness during outbreaks, with special reference to Ebola. BMJ. 2015;351:h3740 10.1136/bmj.h3740 26215666 PMC4516343

[4] Henao-Restrepo AM, Longini IM, Egger M, Dean NE, Edmunds WJ, Camacho A, et al Efficacy and effectiveness of an rVSV-vectored vaccine expressing Ebola surface glycoprotein: interim results from the Guinea ring vaccination cluster-randomised trial. The Lancet. 2015;386(9996):857–866. 10.1016/S0140-6736(15)61117-5 26248676

[5] Geddes AM. The history of smallpox. Clinics in dermatology. 2006;24(3):152–157. 10.1016/j.clindermatol.2005.11.009 16714195

[6] Kretzschmar M, Van den Hof S, Wallinga J, Van Wijngaarden J. Ring vaccination and smallpox control. Emerging infectious diseases. 2004;10(5):832–841. 10.3201/eid1005.030419 15200816 PMC3323203

[7] Halloran ME, Longini IM, Nizam A, Yang Y. Containing bioterrorist smallpox. Science. 2002;298(5597):1428–1432. 10.1126/science.1074674 12434061

[8] Merler S, Ajelli M, Fumanelli L, Gomes MF, y Piontti AP, Rossi L, et al Spatiotemporal spread of the 2014 outbreak of Ebola virus disease in Liberia and the effectiveness of non-pharmaceutical interventions: a computational modelling analysis. The Lancet Infectious Diseases. 2015;15(2):204–211. 10.1016/S1473-3099(14)71074-6 25575618 PMC4409131

[9] Ajelli M, Parlamento S, Bome D, Kebbi A, Atzori A, Frasson C, et al The 2014 Ebola virus disease outbreak in Pujehun, Sierra Leone: epidemiology and impact of interventions. BMC Medicine. 2015;13:281 10.1186/s12916-015-0524-z 26607790 PMC4660799

[10] WHO Ebola Response Team. Ebola Virus Disease in West Africa—The First 9 Months of the Epidemic and Forward Projections. N Eng J Med. 2014;371:1481–1495. 10.1056/NEJMoa1411100 PMC4235004 25244186

[11] Nishiura H, Chowell G. Early transmission dynamics of Ebola virus disease (EVD), West Africa, March to August 2014. Euro Surveill. 2014;19:36. 10.2807/1560-7917.es2014.19.36.20894 25232919

[12] Althaus CL. Estimating the Reproduction Number of Ebola Virus (EBOV) During the 2014 Outbreak in West Africa. PLOS Currents Outbreaks. 2014. 10.1371/currents.outbreaks.91afb5e0f279e7f29e7056095255b288 PMC4169395 25642364

[13] Chowell G, Nishiura H. Transmission dynamics and control of Ebola virus disease (EVD): a review. BMC Medicine. 2014;12(1):196. 25300956 10.1186/s12916-014-0196-0 PMC4207625

[14] Althaus CL. Ebola superspreading. The Lancet Infectious Diseases. 2015;15(5):507–508. 10.1016/S1473-3099(15)70135-0 25932579 PMC7158960

[15] Faye O, Boëlle P, Heleze E, Faye O, Loucoubar C, Magassouba N, et al Chains of transmission and control of Ebola virus disease in Conakry, Guinea, in 2014: an observational study. The Lancet Infectious Diseases. 2015;15(3):320–6. 10.1016/S1473-3099(14)71075-8 25619149 PMC4373532

[16] Nyenswah T, Fallah M, Sieh S, Kollie K, Badio M, Gray A, et al Controlling the last known cluster of Ebola virus disease -Liberia, January—February 2015. MMWR Morb Mortal Wkly Rep. 2015;64(18):500–4. 25974635 PMC4584826

[17] Halloran ME, Longini IM, Cowart DM, Nizam A. Community interventions and the epidemic prevention potential. Vaccine. 2002;20(27):3254–3262. 12213394 10.1016/s0264-410x(02)00316-x

[18] Longini IM, Nizam A, Xu S, Ungchusak K, Hanshaoworakul W, Cummings DAT, et al Containing Pandemic Influenza at the Source. Science. 2005;309(5737):1083–1087. 10.1126/science.1115717 16079251

[19] Ferguson NM, Cummings DAT, Fraser C, Cajka JC, Cooley PC, Burke DS. Strategies for mitigating an influenza pandemic. Nature. 2006;442(7101):448–452. 10.1038/nature04795 16642006 PMC7095311

[20] Wells C, Yamin D, Ndeffo-Mbah ML, Wenzel N, Gaffney SG, Townsend JP, et al Harnessing case isolation and ring vaccination to control Ebola. PLoS Negl Trop Dis. 2015;9(5):e0003794 10.1371/journal.pntd.0003794 26024528 PMC4449200

[21] Kucharski AJ, Eggo RM, Watson CH, Camacho A, Funk S, Edmunds WJ. Effectiveness of Ring Vaccination as Control Strategy for Ebola Virus Disease. Emerg Infect Dis. 2016;22(1):105–108. 10.3201/eid2201.151410 26691346 PMC4696719

[22] The DHS Program. Demographic and Health Survey 2007; 2007. http://dhsprogram.com/Publications/Publication-Search.cfm?ctry_id=22&country=Liberia.

[23] Richards P, Amara J, Ferme MC, Kamara P, Mokuwa E, Sheriff AI, et al Social pathways for Ebola virus disease in rural Sierra Leone, and some implications for containment. PLoS Negl Trop Dis. 2015;9(4):e0003567 10.1371/journal.pntd.0003567 25886400 PMC4401769

[24] Lloyd-Smith JO, Schreiber SJ, Kopp PE, Getz WM. Superspreading and the effect of individual variation on disease emergence. Nature. 2005;438(7066):355–359. 10.1038/nature04153 16292310 PMC7094981

[25] World Health Organization. World Health Organization to Review Mercks Investigational Ebola Vaccine for Emergency Use Assessment and Listing; 2015. http://www.mercknewsroom.com/news-release/vaccine-news/world-health-organization-review-mercks-investigational-ebola-vaccine-eme

[26] GAVI The Vaccine Alliance. Ebola vaccine purchasing commitment from Gavi to prepare for future outbreaks; 2016. http://www.gavi.org/Library/News/Press-releases/2016/Ebola-vaccine-purchasing-commitment-from-Gavi-to-prepare-for-future-outbr

[27] Fenner F, Henderson DA, Arita I, Jezek Z, Ladnyi ID, et al Smallpox and its eradication. Geneva: World Health Organization; 1988.

[28] World Health Organization. A research and development blueprint for action to prevent epidemics; 2016. http://www.who.int/csr/research-and-development/en/.

