# Supplementary material for: Containing Ebola at the Source with Ring Vaccination
Source: PLoS Negl Trop Dis. 2016 Nov 2;10(11):e0005093. doi: 10.1371/journal.pntd.0005093 (PMC5091901; doi:10.1371/journal.pntd.0005093)
Supplement: S1 File — Supporting text containing methodological details and additional results. (PDF) [file pntd.0005093.s001.pdf]

Containing Ebola at the source with ring vaccination

Stefano Merler[1], Marco Ajelli,[1], Laura Fumanelli[1], Stefano Parlamento[1],
Ana Pastore y Piontti[2], Natalie E. Dean[3], Giovanni Putoto[4], Dante Carraro[4],
Ira M. Longini Jr.[3], M. Elizabeth Halloran[5,6], Alessandro Vespignani[2,7,8]

[1]Bruno Kessler Foundation, Trento, Italy
[2]Laboratory for the Modeling of Biological and Socio-technical Systems,
Northeastern University, Boston, MA 02115, USA
[3]Department of Biostatistics, College of Public Health and Health Professions,
University of Florida, Gainesville, FL 32611, USA
[4]Doctors with Africa-CUAMM, Padua, Italy
[5]Vaccine and Infectious Disease Division, Fred Hutchinson Cancer Research Center,
Seattle, WA 98109, USA
[6]Department of Biostatistics, University of Washington, Seattle, WA 98195, USA
[7]Institute for Quantitative Social Sciences at Harvard University, Cambridge, MA 02138, USA
[8]Institute for Scientific Interchange Foundation, Turin, Italy

Contents

1 Material and Methods **3**

1.1 Population model . . . 3

1.2 EVD transmission model . . . 3

1.2.1 General structure . . . 3

1.2.2 Detailed EVD transmission model . . . 6

1.3 EVD transmission model without interventions: effective reproduction number . 7

1.4 EVD transmission model without interventions: model calibration . . . 8

1.5 EVD transmission model with interventions: case isolation and ring vaccination . 10

1.6 EPP and overall vaccine effectiveness: statistical analysis . . . 10

2 Results **11**

2.1 Sensitivity of EPP to values of parameters regulating EVD transmission and interventions . . . 11

2.1.1 Uncertainty regarding preparedness and capacity of the health system. . . 12

2.1.2 Uncertainty regarding the impact of ring vaccination. . . . 13

2.1.3 Spatial vaccination. . . . 13

2.1.4 Variability in EVD transmission. . . . 14

2.1.5 Mortality rate . . . 16

2.2 Baseline ring vaccination: OVE . . . 16

2.3 Sensitivity of OVE to values of parameters regulating EVD transmission and interventions . . . 18

2.4 Vaccine doses and number of rings . . . 19

2.5 Outbreak containment: C&CC+S and "improved health systems" . . . 21

Bibliography **21**

1 Material and Methods

1.1 Population model

We developed a spatial population model of Pujehun district by simulating a population of 375,000 individuals. Individuals were co-located in households. The distribution of the household size was obtained from DHS data [1] (Fig. S1B). Households were aggregated in villages and one major rural town (Pujehun, 30,000 inhabitants). The distribution of the number of households in the villages was determined by the analysis of aerial images (Fig. S1A). Villages were randomly spatially located in the district (see inset of Fig. S1B). Households in Pujehun town and villages were spatially located by assuming that households cover an area of about 15×15 m^2 (as resulting from the analysis of aerial images). Each household was linked to four households (Fig. S1C) mimicking the concept of extended household typical of the rural African context. The extended household defines the set of contacts (and contact of contacts) that can be identified through contact tracing procedures. We set the number of additional households to four to match the size of vaccination rings in [2]: mean simulated ring size: 87.2, 95%CI: 60.8-136.0, mean ring size in [2]: 85.0. The four additional households were randomly chosen with probability decreasing with the geographical distance. Dependence on distance is regulated by information on social pathways in Sierra Leone [3]. Specifically, for each household i, we define a set of weights

$$k_{ij} = k(d_{ij}) = 1/(1 + d_{ij}^b),$$

where d_{ij} is the distance of household i from all households j in the population and parameter b regulates the decrease of the weight with distance (Fig. S1C). Additional households of household i are randomly chosen from the entire set of households in the district with probability proportional to k_{ij}, namely $p_j = k_{ij}/\sum_l k_{il}$. The value of parameter b was selected in order to match data on the distance of socio-economical connections between villages in Sierra Leone (7.7 km on average [3]). The resulting optimal value of parameter is $b = 2.25$ (Fig. S1D). The resulting cumulative distribution of the distance of additional households is reported in the inset of Fig. S1D. Note that location of additional households depends on both geographical distance and population density. We also discussed scenarios by assuming different values of b, namely $b = 4$ resulting in an average distance of 1.5 km, $b = 3$ resulting in an average distance of 3.5 km, $b = 1.5$ resulting in an average distance of 15 km, $b = 1$ resulting in an average distance of 25 km. We divided the population into two age classes, namely children (≤ 15 years old, corresponding to 48.3% of the population) and adults.

1.2 EVD transmission model

1.2.1 General structure

The 2014 EVD outbreak in Pujehun district was mainly determined by contacts in households (35.9% of transmission), in extended households (38.5% of transmission), random contacts in the population (mostly among friends, 17.9% of transmission), and contacts in health care facilities (7.7% of transmission – of which 67% from infected patients to health care workers) [4]. Above proportions account for potential transmission events during burial ceremonies (88.1% of fatal

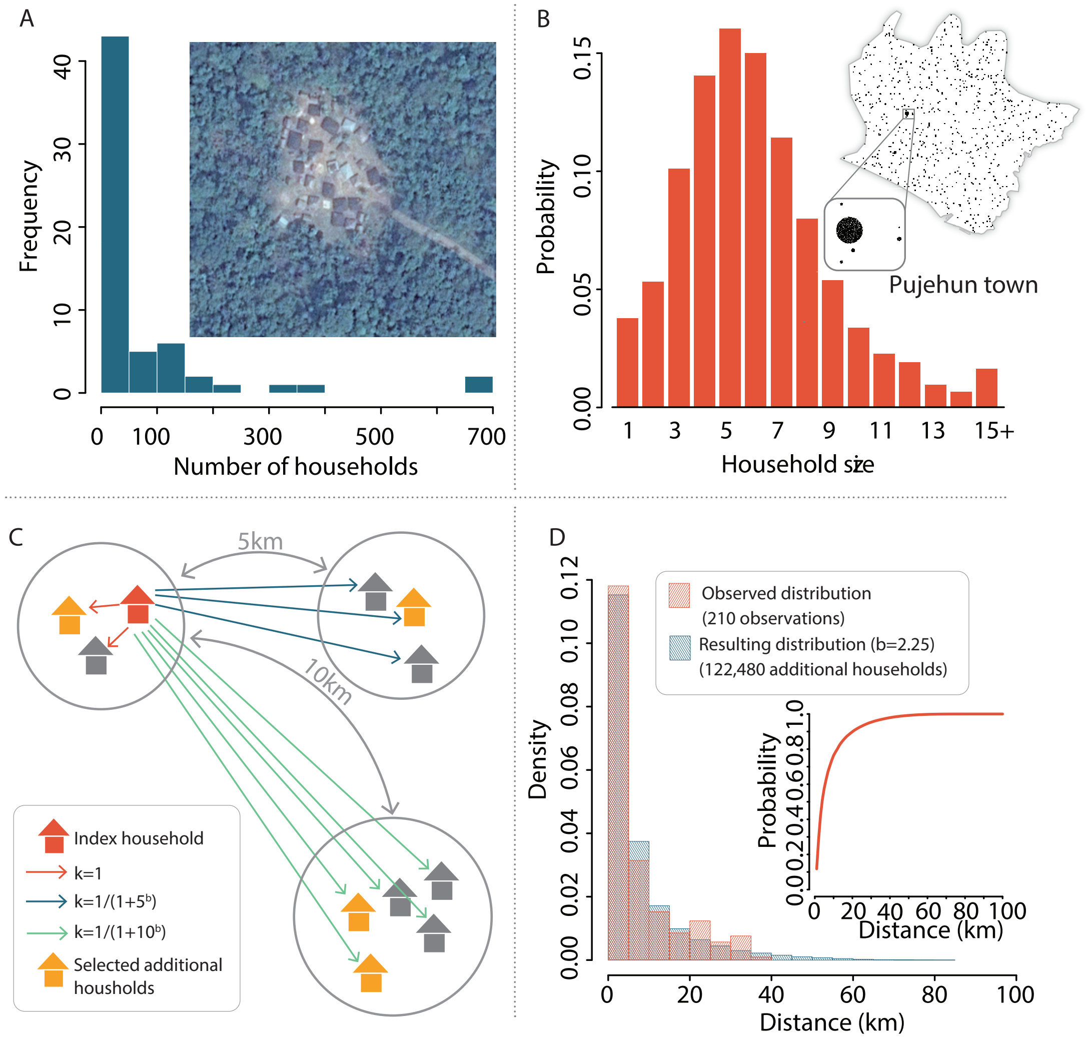

Figure S1: Population model of Pujehun district. **A** Distribution of the number of households in villages of Pujehun district. **B** Distribution of household size in Sierra Leone. **C** Schema of the procedure for selecting additional households in a simulated population with three villages. For each household, additional households are randomly sampled from the entire set of households in the population with probability proportional to a kernel function $w(d) = 1/(1+d^b)$ decreasing with the geographical distance. **D** Comparison between the observed distribution of the distance of socio-economical connections between villages in Sierra Leone [3] (red) and the distribution of the distance of additional households from index households (only additional households not in the same village of index households are considered) with $b = 2.25$ (blue). Cumulative probability of the distance of extended households from index households is shown in the inset.

cases were buried safely [4]), and compare well with estimates for Guinea at the beginning of

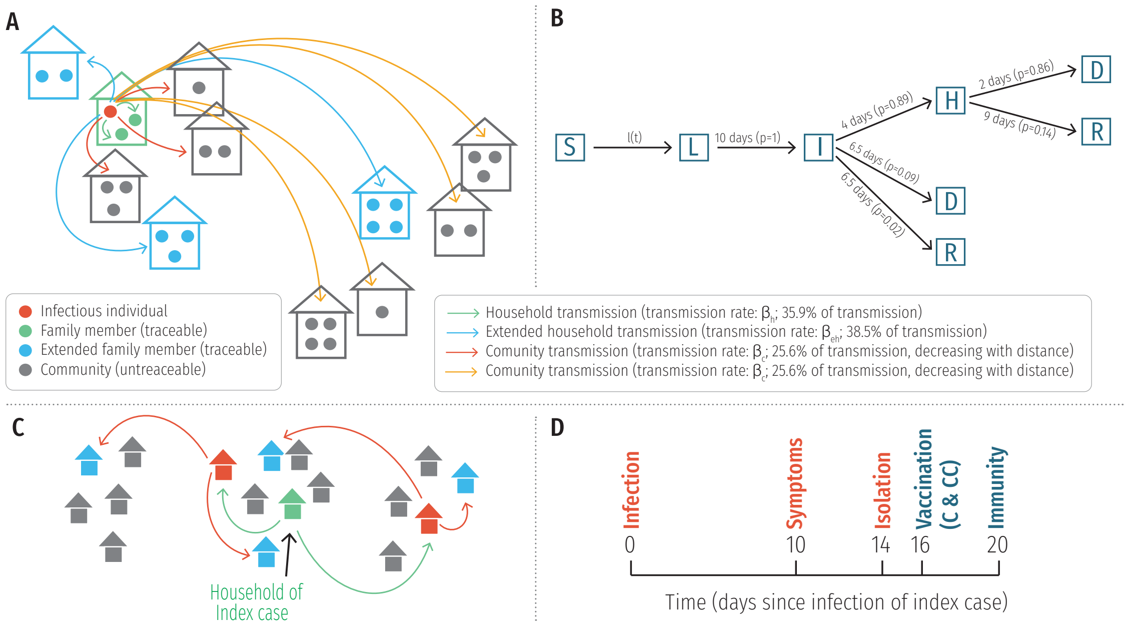

Figure S2: Transmission model. **A** Schema of transmission model with explicit transmission in households and extended households (only 3 extended households in 2 villages are shown) and distance-dependent transmission in the general community. **B** Class S represents susceptible individuals, class L represents latent individuals, class I represents infectious symptomatic individuals, class H represent hospitalised individuals, class R represent recovered individuals, and class D represents deceased individuals. Durations (in days) and probabilities of transition are shown. **C** Schema of ring vaccination by assuming 3 villages: once a patient is isolated the ring is defined as contacts of index cases and contacts of contacts. Contacts are defined as people living in the same household of index case (green household) and in the extended household of index case (red households). Contacts account for 74.4% of transmission. Contacts of contacts are represented by blue households. **D** (Mean) Key time periods regulating progression of EVD (red) and ring vaccination (blue).

the EVD epidemic [5], and with model estimates obtained for Liberia [6].

We simulated the spread of EVD in Pujehun district by making use of a spatially explicit microsimulation model accounting for the explicit transmission in households and extended households (35.9% and 38.5% of transmission, respectively; 74.4% of transmission overall), and in the general community, accounting for all other contacts, e.g. other contacts in the population or health care related contacts (25.6% of transmission overall) (Fig. S2A). This structure allows us to identify the contacts that can be targeted by ring vaccination policies through contact tracing, namely contacts in households and extended households, and contacts of contacts [2] (Fig. S2C). Transmission in the general community considers that interactions decays with the distance because of the reduced likelihood of long-range mobility [3] (Fig. S1D). Moreover, we assume that in the event of a new outbreak, health care workers (HCW) would be promptly

immunized, thereby drastically reducing EVD transmission in hospital settings as compared to the 2014-15 EVD epidemic in West Africa [6]. With vaccinated HCWs, hospital transmission would primarily impact Ebola-uninfected individuals seeking health care in hospitals for other illnesses, and this is accounted along with transmission in the general community.

The basic reproduction number of EVD estimated in Pujehun district was $R_0 = 1.67$ [4]. Similar estimates were obtained for other regions of West Africa [7, 8, 9, 10, 6]. However, in a new outbreak, possibly originating in a different region of Africa, the transmission potential could be remarkably different. For instance, it could be either decreased or increased by human behaviour, by the ability of health care workers to limit transmission in health care facilities, by burial procedures. This is the reason why we explored a much wider range of values for the effective reproduction number R_e, ranging from 1.4 to 2.6.

1.2.2 Detailed EVD transmission model

Simulations were initialised with one index case randomly chosen in the population. At the end of the latent period (10 days on average [4]), the index case can transmit the infection through contacts in household, with members of the extended family and in the general community. We assume no asymptomatic transmission and that all cases develop clinical symptoms. Under the assumption of homogeneous mixing, at time t, a relative j of the index case i is exposed to a force of infection

$$\lambda_j(t) = \beta_h \rho_i / n(H_{jt}),$$

where β_h is the transmission rate in household, ρ_i is the infectiousness of the index case, and $n(H_{jt})$ is the household size at time t – household size can vary because members die or are hospitalised. ρ_i is drawn from a Gamma distribution of mean 1 and shape 0.45 to account for the observed heterogeneity in transmission (see for instance [11]). In this way the number of secondary cases per primary infector is distributed according to a negative binomial distribution of dispersion parameter 0.45, as observed in Pujehun district [4]. A similar equation regulates transmission to members of the extended family (with transmission rate β_{eh}). As for the transmission in the general community, any individual j is exposed to a force of infection decreasing with the distance, namely

$$\lambda_j = \beta_c \rho_i k(d_{ji}) / \sum_l k(d_{il}),$$

where β_c is the transmission rate in the general community, $k(d)$ is the distance kernel defined above, d_{ji} is the geographical distance between individuals i and j. This is a procedure similar to that used for describing the spatial transmission of influenza in the general community [12, 13, 14, 15]. The index case is hospitalised with probability 88.8%. In case of hospitalization, transmission in the community lasts 4 days on average [4], 6.5 days on average otherwise [4]. The case fatality rate is set to 85.7% [4]. If hospitalised, the index case remains in hospital 2 days on average in case of death, 9 days on average in case of recovery [4]. The simulated epidemic is generated by applying the above described process to all newly infected individuals (Fig. S2B).

All in all, at any time t of the simulation, a susceptile individual j is exposed to the following

force of infection:

$$\lambda_j(t) = \sum_{i \in H_{jt}} \frac{I_i \beta_h \rho_i}{n(H_{jt})} + \sum_{i \in EH_{jt}} \frac{I_i \beta_{eh} \rho_i}{n(EH_{jt})} + \sum_{i=1}^{N} \frac{I_i \beta_c \rho_i k(d_{ji})}{\sum_{l=1}^{N} k(d_{il})},$$

where $I_i = 1$ if individual i is infectious and $I_i = 0$ otherwise; N is the population size; H_{jt} is the set of individuals in the same household of individual j at time t and $n(H_{jt})$ is its cardinality at time t (it varies over time because of deceased and hospitalised cases); EH_{jt} is the set of individuals in the expended household of individual j at time t and $n(EH_{jt})$ is its cardinality at time t.

The probability of getting infected upon exposure is

$$p_j(t) = \sigma(1 - \exp\{\lambda_j(t)\Delta t\}),$$

where $\sigma = 1$ for adults and $\sigma = 0.247$ for children to account for the age specific risk of infection observed in Pujehun district [4], and $\Delta t = 1$ day is the time step of the simulation.

1.3 EVD transmission model without interventions: effective reproduction number

According to the definition given in [16], R_e (as stated above, we prefer talking of effective rather than basic reproductive number, as transmissibility of EVD might be affected by several factors) of an epidemic is the solution of the equation

$$1 = R_e \int_0^\infty \gamma(\tau) e^{-r\tau} d\tau,$$

where r is the exponential growth rate of the simulated epidemic during the initial phase and $\gamma(\tau)$ is the distribution of the generation time of the model. Such a definition has been used in several studies on different infectious diseases [7, 14, 13, 17, 18, 19].

In Fig. S3A the distribution (10,000 model realizations with R_e=1.6) of the number of secondary cases generated by the index cases is shown. As a consequence of transmission heterogeneity that we have considered in the model in order to reproduce the data on the EVD outbreak in Pujehun [4], this distribution is well fitted by a negative binomial (see Fig. S3B), but not by a Poisson distribution (see Fig. S3C). The mean of the distribution of secondary cases of the index case, commonly referred to as R_{index}, is $R_{index} = 2.0$. Moreover, this distribution has a long tail, with an appreciable probability of up to 10 or more transmissions (see Fig. S3A). By analyzing scenarios assuming $R_e = 1.6$, 2, and 2.4, we found that $R_{index} \approx R_e + 0.4$. The reason is that the model is characterized by very clustered transmission resulting from i) structure of contacts (75% of transmission among close contacts, that is about 30 persons) and ii) spatial structure (villages) [20]. R_{index} is indeed the outcome of a heterogenous transmission process and the lack of a typical index case (difference in the household structure, extended household, village size etc.). To obtain a given exponential growth in cases in the community for the early stage of the outbreak, the model needs transmission rates resulting in a larger number of cases generated by the very first few cases (a few realisations are reported in Fig. S4). The difference between the two estimates of R_e and R_{index} stems from the fact that R_e is estimated over the

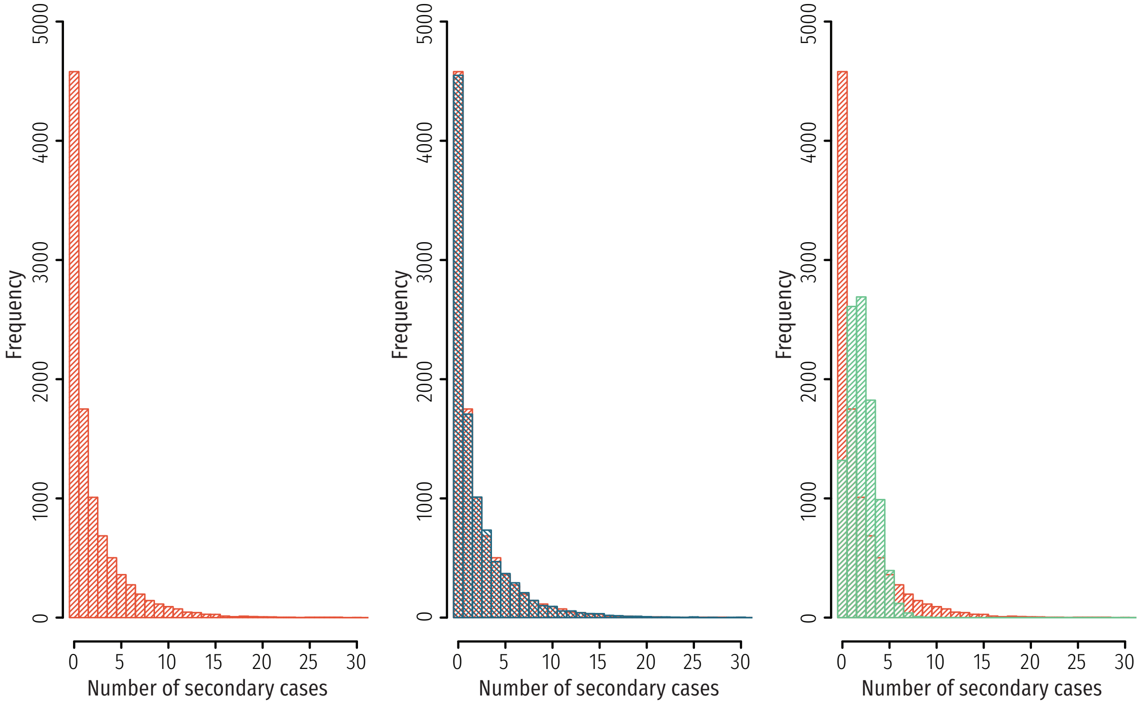

Figure S3: **A** Distribution of the number of secondary cases generated by the index cases for $R_e = 1.6$. **B** Distribution of the number of secondary cases generated by the index cases for $R_e = 1.6$ and negative binomial fit. **C** Distribution of the number of secondary cases generated by the index cases for $R_e = 1.6$ and Poisson fit.

"standard" phase of sustained exponential growth, while R_{index} is estimated by looking at cases generated only by the very first index case.

A similar pattern has been reported for models of influenza transmission [17, 21, 13]. In particular, differences for influenza were slightly lower: $R_{index} = R_e + 0.2$ (while for EVD we obtain a difference of 0.4). This can be explained by considering that transmission in influenza models is less clustered (transmission in the general community is assumed to by about 33% and, more importantly, there are also other settings such as schools and workplaces where an individual can have a large number of contacts). Moreover, in influenza models the distribution of secondary cases is commonly assumed to be Poisson distributed (while for EVD we are assuming a negative binomial distribution). These factors contribute to an earlier saturation of the network of contacts for Ebola transmission models – this does not hold for homogenous models as they do not consider the clustered structure of contacts actually observed [22].

1.4 EVD transmission model without interventions: model calibration

The model has three free parameters, corresponding to the three transmission rates. Calibration was performed by identifying values of the three transmission rates $(\beta_h, \beta_{eh}, \beta_c)$ resulting, on

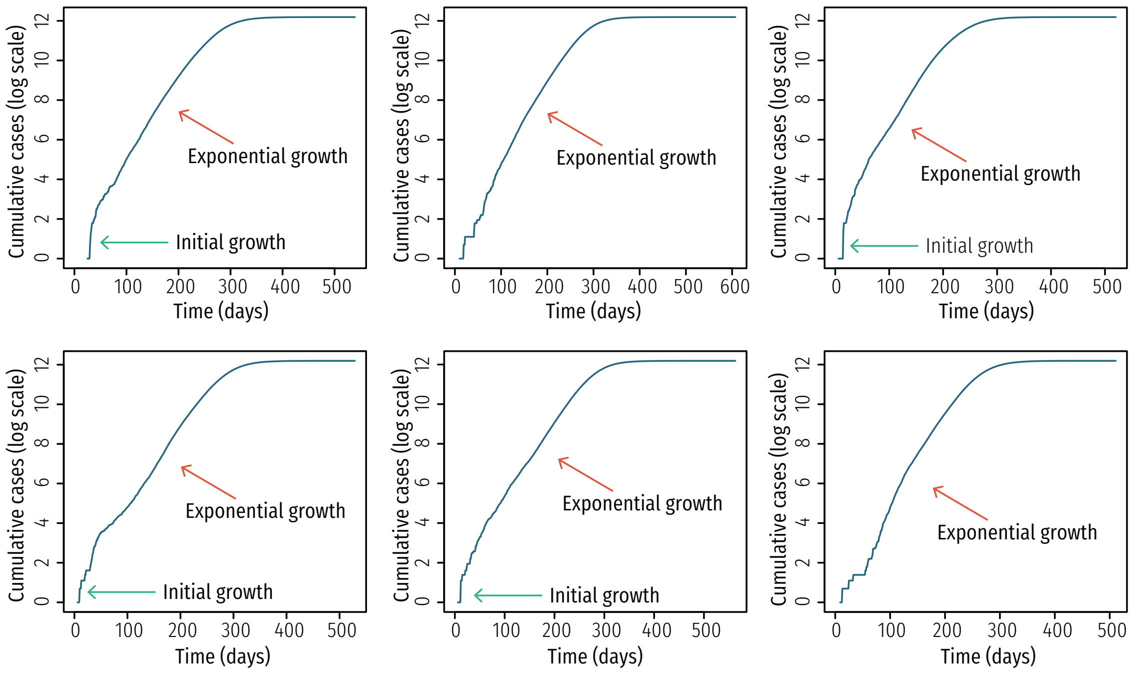

Figure S4: Cumulative number of symptomatic cases (log scale) of 6 randomly selected simulated epidemics with $R_e = 1.6$.

average, in simulated epidemics characterised by $R_e = 1.67$ and such that 35.9% of cases are generated through contacts in the households, 38.5% through contacts in the extend households, and 25.6% through contacts in the general community (aggregation of all non-household and non-extended household transmission events). The following procedure was used:

- define the sampling space as the cube $S = [0, K]^3$ with $K = 1$ (chosen after preliminary inspection);
- create a regular grid in S of N^3 points $(\beta_h, \beta_{eh}, \beta_c)$, with $N = 20$;
- for each point $(\beta_h, \beta_{eh}, \beta_c)$ run 1,000 model simulations initialised with 100 initial cases (to avoid auto-extinction), compute the average R_0 and the average percentage of cases by setting (P_h, P_{eh}, P_c);
- choose the point $(\beta_h^\star, \beta_{eh}^\star, \beta_c^\star)$ with minimum error in terms of R_0 (target value: 1.67) and percentage of cases by setting (P_h, P_{eh}, P_c) (target vales: 35.9%, 38.5%, 25.6%);
- redefine S as the cube centered in $(\beta_h^\star, \beta_{eh}^\star, \beta_c^\star)$ and $K = K/3$, consider only points belonging to $[0, 1]^3$, and reapply the above described procedure;
- iterate until convergence is obtained.

The resulting values of the three transmission rates are: $\beta_h^\star = 0.331$ days^{-1}, $\beta_{eh}^\star = 0.054$ days^{-1} and $\beta_c^\star = 0.109$ days^{-1}. As stated above, we are interested to explore a much wider range of value of R_e, ranging from 1.4 to 2.6. These scenarios were obtained by multiplying the three transmission rates $(\beta_h^\star, \beta_{eh}^\star, \beta_c^\star)$ by the proper proportionality factor to obtain the desired value of R_e. Note that the model was calibrated to generate the proportions of cases per setting in Pujehun district with $R_e = 1.67$ (the value observed in the Pujehun district). When we change R_e (by modifying the transmission rates), the proportions of cases by setting may change as well.

1.5 EVD transmission model with interventions: case isolation and ring vaccination

We assume that the epidemic can be detected after a certain number of symptomatic cases (5 cases in baseline simulations). As soon as the outbreak is detected, newly hospitalized cases are isolated (we assume hospitalization at 88.8% and the availability of 20 Ebola beds in baseline simulations [4]) and trigger ring vaccination. Individuals eligible for vaccination are contacts of index cases, and contacts of contacts [2]. Note that when we explore scenarios with a lower number of Ebola beds (e.g. 1 to 5), sick individuals seek assistance but, when no beds are available, cases are not hospitalized (i.e., <88.8% hospitalized) and thus continue to transmit the infection in the community. In the baseline simulations we assume a vaccine coverage of 65% [2] and vaccine efficacy equal to 90% [2]. Moreover, we assume 2 days on average to deliver the vaccine and 4 days on average for the vaccine to develop protective immunity (overall, 6 days on average for the vaccine to develop protective immunity from ring enrollment). Key time periods regulating infection transmission and vaccination process are reported in Tab. 1 of the main text and summarized in Fig. S2B and Fig. S2D.

1.6 EPP and overall vaccine effectiveness: statistical analysis

We measure the impact of ring vaccination on simulated outbreaks in terms of the epidemic prevention potential (EPP). The EPP is defined as EPP$= 1 - p_I/p_{NI}$, where p_I and p_{NI} are the probabilities of an uncontained outbreak when an intervention is used (e.g. ring vaccination) or no intervention is used, respectively. Here an outbreak is considered as uncontained if the number of cases exceeds 300, though the results are consistent if this threshold is varied by up to 30%. Note that EVD had never resulted in more than 425 cases (Uganda outbreak in 2000) before the 2014-15 epidemic in West Africa. Confidence intervals of EPP were computed by assuming that the number of uncontained outbreaks with and without intervention has a binomial distribution, $B(p_I, n)$ and $B(p_{NI}, n)$ respectively:

$$1 - \frac{p_I}{p_{NI}} e^{\pm\Phi^{-1}(\alpha/2)\sqrt{\frac{1-p_I}{np_I}+\frac{1-p_{NI}}{np_{NI}}}},$$

where Φ^{-1} denotes the normal quantile function and $n = 1000$ is the number of model realizations. The EPP indicator is discussed in the main text.

Another valuable public health measure is the number of averted cases in medium-long term because it quantifies the impact of the intervention in reducing the overall disease burden.

Providing estimates for this indicator is quite challenging as it requires to know in advance which non-pharmacological interventions will be considered (in the absence of an effective vaccine) and their possible impact. Behavioural changes of the population are impossible to quantitatively predict as well. For these reasons, here we analyse overall vaccine efficacy (OVE) with respect to the worst case scenario, where we assume no interventions and no behavioural changes in the population. As observed during the 2014-15 EVD epidemic in West Africa, this is a fairly unrealistic scenario and thus results should be considered cautiously. The proportional reduction in cases is called the overall vaccine effectiveness, defined as OVE$= 1 - AR_I/AR_{NI}$, with AR_I and AR_{NI} denoting the population attack rate one year after emergence of the first case (i.e., the ratio between the cumulative number of cases after one and the total population), regardless of whether the outbreak is contained or not, when interventions are in place or not, respectively.

The standard deviation of the OVE indicator was computed by using standard equations for calculating propagation of uncertainty for non-linear combinations of random variables, namely:

$$\sqrt{\left(\frac{\partial f}{\partial x}\right)^2 \sigma^2(x) + \left(\frac{\partial f}{\partial y}\right)^2 \sigma^2(y)},$$

where $x = AR_I$, $y = AR_{NI}$, $f(x, y) = 1 - x/y$, σ denotes the standard deviation, and partial derivatives are evaluated at the mean value of all components x and y.

2 Results

2.1 Sensitivity of EPP to values of parameters regulating EVD transmission and interventions

Key model parameters and their assumed values are summarized in Table 1 in the main text. As baseline values, we assume that a new Ebola outbreak is detected after five symptomatic cases occur. As soon as the outbreak is identified, 88.8% of new cases are isolated [4] and hospitalized an average of 4 days after symptom onset [4]. We assume the availability of 20 Ebola beds [4]. Note that when we explore scenarios with a lower number of Ebola beds (e.g. 1 to 5), sick individuals seek assistance but, when no beds are available, cases are not hospitalized (i.e., <88.8% hospitalized) and thus continue to transmit the infection in the community. Case isolation and hospitalization triggers ring vaccination of contacts (C) and contacts of contacts (CC) for any case who is not already part of an existing vaccination ring [2]. From the time of case isolation, we assume 2 days are required to define the ring, obtain consent from ring members, and administer vaccine, followed by an additional 4 days for vaccinees to develop protective immunity [2]. Vaccine is delivered to 65% of the ring members [2], and vaccine efficacy is conservatively assumed to be 90% [2].

An extensive analysis is performed to assess sensitivity of results to all parameters regulating EVD transmission and interventions. Regarding preparedness and capacity of the healthcare system, we vary assumptions for the time required to detect the outbreak, hospitalization rate, and the number of available Ebola beds. Regarding ring vaccination, we vary assumptions for the vaccine efficacy, vaccine coverage, time for the vaccine to develop protective immunity, and definition of the eligible population. Regarding EVD transmission, we vary assumptions for the

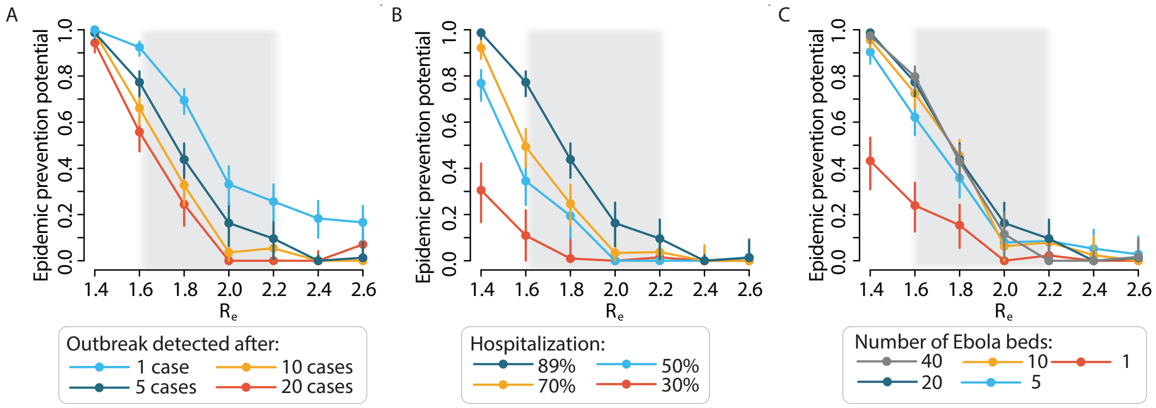

Figure S5: Preparedness and response of the health system. **A** Estimated EPP (points) and 95%CI (vertical lines) as a function of R_e and by varying the number of cases required to detect the outbreak. Baseline value: 5 cases. The shaded grey area represents the range of most plausible values of R_e for the 2014-15 epidemic in West Africa. **B** As in **A** but by varying the percentage of hospitalized cases. Baseline value: 88.8% [4]. **C** As in **A** but by varying the number of Ebola beds. Baseline value: 20 Ebola beds [4]. Each point estimate is based on the analysis of 1,000 simulated epidemics.

time from symptom onset to hospitalization, heterogeneity in transmission, human mobility, and percentage of traceable contacts. We also compare the impact of ring vaccination with preemptive mass vaccination strategies and reactive spatial vaccination strategies. Specifically for reactive spatial vaccination, we consider spatial rings of increasing radius, mimicking the increasing effect of targeting the village of index case, the nearest few villages around the index cases, up to targeting the chiefdom of index case (the Pujehun district is made up of 12 chiefdoms).

2.1.1 Uncertainty regarding preparedness and capacity of the health system.

The delay in implementing ring vaccination is very critical. If the outbreak is detected after 20 EVD cases (roughly corresponding to 6 generations of transmission if $R_e = 1.6$), EPP drastically decreases and containment is uncertain for values of R_e as low as 1.6 (EPP≈ 0.6) (Fig. S5A). On the contrary, EPP is close to 40% even for $R_e = 2$ if the outbreak is readily detected (Fig. S5A). Another critical factor is the capacity of isolating cases. In baseline ring vaccination we assume hospitalization of EVD cases at 88.8%, as observed in Pujehun district in July-October 2014 [4]. However, the percentage of isolated EVD cases in West Africa during the same period was 52% (as reported by the WHO on November 5, 2014 [23]), while the target set by the WHO by 1 December 2014 was 70%. Fig. S5B shows that if EVD cases are isolated at a rate of 30-50%, EPP is very low when $R_e = 1.6$.Fig. S5C shows that EPP does not increase much with the availability of more than 10 Ebola beds. These results, even in light of the response delay during the 2014-15 EVD epidemic in West Africa, claim for a reinforcement of the surveillance systems

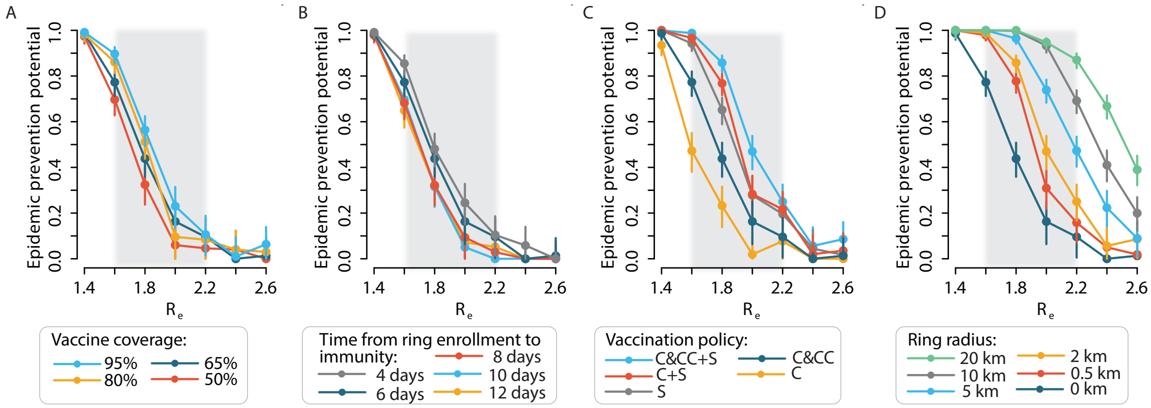

Figure S6: Ring vaccination policy. **A** Estimated EPP (points) and 95%CI (vertical lines) as a function of R_e and by varying the vaccine coverage. Baseline value: 65% [2]. The shaded grey area represents the range of most plausible values of R_e for the 2014-15 epidemic in West Africa. **B** As in **A** but by varying the time for the vaccine to develop protective immunity from ring enrolment. Baseline value: 6 days [2]. **C** As in **A** but by varying the eligible population. Symbols: C indicates contacts of index cases; CC indicates contacts of contacts; S indicates geographical ring (ring radius: 2 km). Baseline value: C&CC [2]. **D** As in **A** but by varying the radius of the geographical ring. Baseline value: 0 km (no spatial ring). Each point estimate is based on the analysis of 1,000 simulated epidemics.

in order to guarantee a rapid response to an emerging outbreak. Moreover, it is necessary to improve the ability to rapidly identify and isolate EVD cases because isolation of cases is the primary intervention for reducing transmission within the community.

2.1.2 Uncertainty regarding the impact of ring vaccination.

In baseline ring vaccination we assume that vaccine is delivered to 65% of the eligible population, corresponding to the percentage of consenting adults observed during the 2015 ring vaccination trial [2]. Later, however, vaccination was extended also to children [2]. Thus, it is likely that vaccine uptake would increase during future EVD outbreaks, also because vaccine safety has been demonstrated [2]. We estimated that EPP is larger than 50% even for $R_e = 1.8$ if coverage is no lower than 80% (Fig. S6A). Number of rings and vaccine doses do not increase substantially, at least for $R_e < 2$, by assuming 95% vaccine coverage (see Fig. S13). If the time needed to define the ring and deliver vaccine increases from 0 to 8 days on average (we assume an additional 4 days on average for the vaccine to develop protective immunity), large drops in EPP are observed only in a restricted range of values of R_e, namely 1.6-2 (Fig. S6B).

2.1.3 Spatial vaccination.

Fig. S6C shows that adding a spatial component to the vaccination ring (here we assume a ring radius of 2 km) would be crucial to increase the EPP. Indeed, vaccination of 65% of C&CC

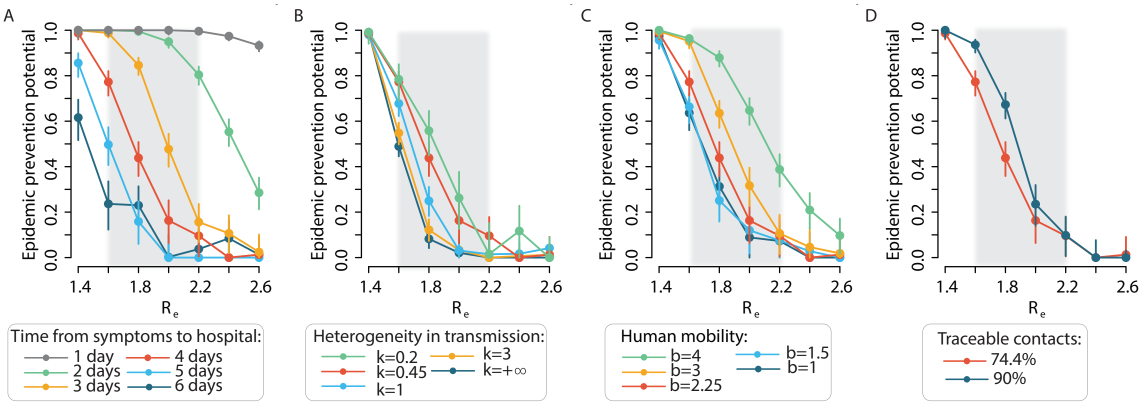

Figure S7: EVD transmission characteristics. **A** Estimated EPP (points) and 95%CI (vertical lines) as a function of R_e and by varying the time from symptoms onset to hospitalization. Baseline value: 4 days [4]. The shaded grey area represents the range of most plausible values of R_e for the 2014-15 epidemic in West Africa. **B** As in **A** but by varying the time for the dispersion parameter regulating transmission heterogeneity. The number of secondary infections has a negative binomial distribution. Baseline value of the dispersion parameter: $k = 0.45$ [4]. $k = 0.2$ is the dispersion parameter estimated in [11] for Conakry, Guinea. $k = +\infty$ corresponds to homogeneous, Poisson transmission. In this scenarios, probabilities of outbreak in the absence of interventions were recomputed for each value of the dispersion parameter k. **C** As in **A** but by varying the parameter regulating human mobility. Spatial transmission is proportional to a power low kernel $1/(1 + d^b)$ where d is the geographical distance and b regulates the decrease of transmission with distance. The resulting average distance of socio-economical relationships is reported between square brackets. Baseline value: $b = 2.25$, resulting in an average distance of 7.7 km [3]. In this scenarios, probabilities of outbreak in the absence of interventions were recomputed for each value of the parameter b. **D** As in **A** but by varying the percentage of traceable cases. Baseline value: 74.4%. Each point estimate is based on the analysis of 1,000 simulated epidemics.

(accounting for about 75% of the transmission [4]) is equivalent to targeting only about 50% of potential second generation contacts. This would be the minimal percentage in case of proactive mass vaccination for preventing an EVD epidemic if $R_e = 2$ and thus it is insufficient with a ring vaccination policy. For spatial ring vaccination strategies, we explored varying the ring radius from 0.5 km (roughly corresponding to enrolling people in the village of index case) to 20 km (roughly corresponding to immediate mass vaccination of a large part of the district). We observed a drastic increase in EPP for all values of R_e larger than 1.6 (Fig. S6D).

2.1.4 Variability in EVD transmission.

In baseline simulations, we assume that transmission in the community lasts 4 days on average [4]. Fig. S7A show that increasing this period, for instance because of sustained transmission

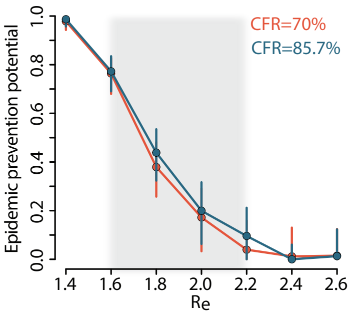

Figure S8: **A** Estimated EPP (points) and 95%CI (vertical lines) as a function of R_e and by varying the CFR. Baseline value: 85.7% [4]. The shaded grey area represents the range of most plausible values of R_e for the 2014-15 epidemic in West Africa.

in hospital settings or during burial ceremonies, or decreasing it, for instance because of the implementation of additional control measures (e.g. the time from symptoms onset to hospitalization has been reduced to two days on average in the last part of the epidemic in West Africa [7] as an effect of interventions, e.g. contact tracing) may drastically affect EPP. Specifically, if the transmission in the community is reduced to two days on average, EPP is larger than 0.5 for R_e as large as 2.4. Here we stress that ring vaccination requires identification of contacts (and contacts of contacts). However, identification alone does not necessarily imply early isolation of potential secondary cases. Increasing this period up to 6 days may make it unlikely to contain EVD outbreaks with R_e as low as 1.6. In agreement with results reported in [24], we found that heterogeneity in transmission, a pattern observed both in Sierra Leone [4] (65% of cases did not transmit the infection) and Guinea [11], might be key in determining a successful containment (Fig. S7B). The reasons for the observed pattern are not clearly understood yet. In baseline ring vaccination, we assume an average distance of socio-economical relationships of about 7.7 km [3]. We found that human mobility may strongly impact on the outcome of ring vaccination (Fig. S7C). This is explained by considering that decreasing the mobility of people results in a more clustered transmission, thus increasing the probability of outbreak containment. It should be noted, however, that changing the parameter regulating mobility b does not affect much the probability of outbreak in the absence of intervention. Indeed, by model construction, variations of b modify the distance of secondary cases in the general community, while the number of secondary cases remains nearly constant. In other words, we do not assume any reduction in the time spent in the community or in the number of random contacts in the population. Changes of b only modify the probability that secondary cases in the general community belong to the network of C&CC targeted by ring vaccination. In baseline ring vaccination we assume that about 75% of contacts of EVD cases are detectable through contact tracing [4]. However, in [4], authors found that a certain fraction of EVD cases not infected through family contacts could be detected as well. In Fig. S7D we analyse the impact of baseline interventions by assuming that the fraction of contacts detectable through contact tracing is 90%. It emerges that the EPP increases substantially in the critical range of R_e between 1.6 and 2.

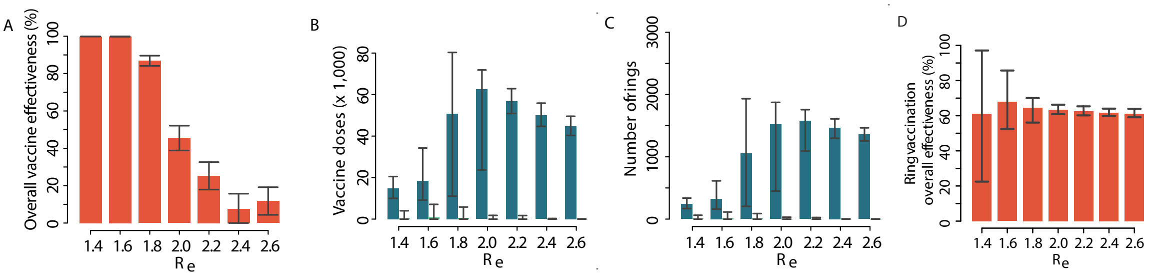

Figure S9: **A** Estimated overall vaccine effectiveness ($\pm$ 2SE) one year after the emergence of the first case (with respect to worst-case scenario) as a function of R_e. **B** Mean number of vaccine doses and 95%CIs one year after the emergence of the first case for a range of R_e values. Both contained (green) and uncontained (blue) outbreaks are considered. **C** As B but for the number of rings defined. . The number of rings and vaccine doses used in the first year decreases at very high R_e. This is explained by considering that the maximum number of rings that can be defined in one year is constrained by the capability of the health systems and epidemics with high R_e may terminate within 1 year since the first case. **D** Mean effectiveness of ring vaccination and 95%CI. Each estimate is based on the analysis of 1,000 simulated outbreaks.

2.1.5 Mortality rate

In baseline ring vaccination we assume a case fatality rate at 85.7%. This is the average value observed in Pujehun district during the 2014-15 EVD epidemic, namely 42/49 (95% CI: 72.7-94.1) [4]. This value is higher than that estimated on larger population samples, namely about 70% [7]. However, as shown in Fig. S8, results are not sensitive to value of the assumed CFR. Moreover, larger CFR at the very beginning of the outbreak are possible, especially prior to outbreak detection, as documented in previous outbreaks [25, 26].

2.2 Baseline ring vaccination: OVE

The EPP of ring baseline vaccination is discussed in the main text (Fig. 1). Even when the outbreak is not fully contained by ring vaccination, the OVE is high ($>$ 85%) for values of $R_e \leq 1.8$ (Fig. S9A). The OVE when $R_e = 1.8$ is similar to the 75.1% OVE (95%CI: -7.1-94.2) observed in the ring vaccination trial in Guinea [2]. On average, 326 rings and 18,445 vaccine doses are required in the first year of an outbreak when $R_e = 1.6$, if the outbreak is not readily contained (Fig. S9B,C). Moreover, we computed ring vaccination effectiveness by assuming that only 50% of enrolled rings are vaccinated and by comparing attack rates of vaccinated versus non-vaccinated rings. By assuming baseline interventions (Fig. 1 in the main text), the estimated overall effectiveness of ring vaccination is about 65%-70% (Fig. S9D), similar to the estimate reported in [2], namely 75.1%. These results suggest that ring vaccination strategies can be highly effective and affordable for containing an EVD outbreak when $R_e \leq 1.6$ and can greatly decrease case burden for uncontained outbreaks when $R_e \leq 2.0$.

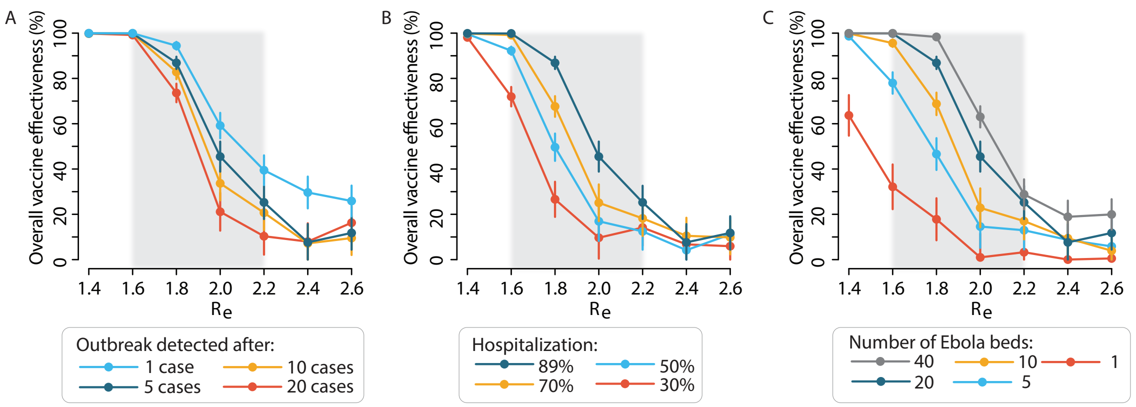

Figure S10: Preparedness and response of the Health system. **A** Estimated OVE (± 2 SE) one year after the emergence of the first case (with respect to worst case scenario) as a function of R_e and by varying the number of cases required to detect the outbreak. **B** As in **A** but by varying the percentage of hospitalized cases. Baseline value: 88.8% [4]. **C** As in **A** but by varying the number of Ebola beds. Baseline value: 20 Ebola beds [4]. Each point estimate is based on the analysis of 1,000 simulated epidemics.

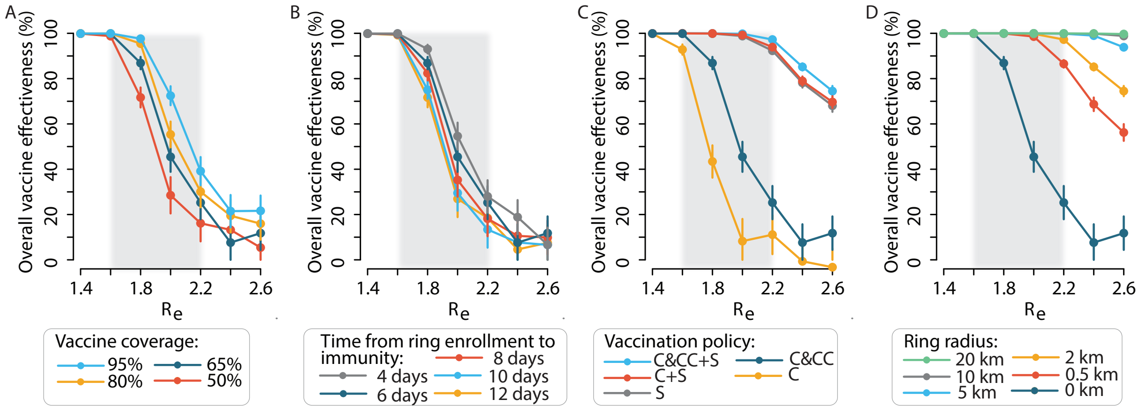

Figure S11: Ring vaccination policy. **A** Estimated OVE (± 2 SE) one year after the emergence of the first case (with respect to worst case scenario) as a function of R_e and by varying the vaccine coverage. **B** As in **A** but by varying the time for the vaccine to develop protective immunity from ring enrolment. Baseline value: 6 days [2]. **C** As in **A** but by varying the eligible population. Symbols: C indicates contacts of index cases; CC indicates contacts of contacts; S indicates geographical ring (ring radius: 2 km). Baseline value: C&CC [2]. **D** As in **A** but by varying the radius of the geographical ring. Baseline value: 0 km (no spatial ring). Each point estimate is based on the analysis of 1,000 simulated epidemics.

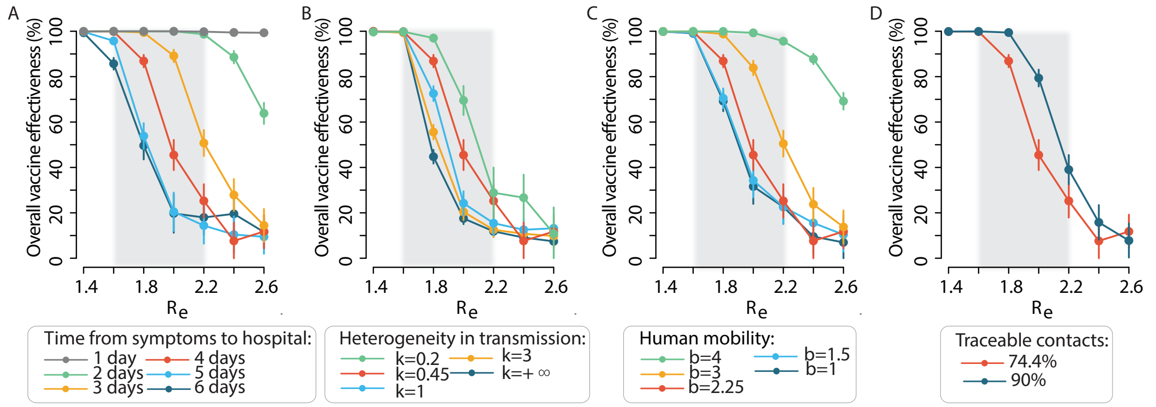

Figure S12: EVD transmission characteristics. **A** Estimated OVE (± 2 SE) one year after the emergence of the first case (with respect to worst case scenario) as a function of R_e and by varying the time from symptoms onset to hospitalization. **B** As in **A** but by varying the time for the dispersion parameter regulating transmission heterogeneity. The number of secondary infections has a negative binomial distribution. Baseline value of the dispersion parameter: $k = 0.45$ [4]. $k = 0.2$ is the dispersion parameter estimated in [11] for Conakry, Guinea. $k = +\infty$ corresponds to homogeneous, Poisson transmission. In these scenarios, probabilities of outbreak in the absence of interventions were recomputed for each value of the dispersion parameter k. **C** As in **A** but by varying the parameter regulating human mobility. Spatial transmission is proportional to a power law kernel $1/(1+d^b)$ where d is the geographical distance and b regulates the decrease of transmission with distance. Baseline value: $b = 2.25$, resulting in an average distance of 7.7 km [3]. In these scenarios, probabilities of outbreak in the absence of interventions were recomputed for each value of the parameter b. **D** As in **A** but by varying the percentage of traceable cases. Each point estimate is based on the analysis of 1,000 simulated epidemics.

2.3 Sensitivity of OVE to values of parameters regulating EVD transmission and interventions

In Fig. S10 we report results of a sensitivity analysis, similar to that conducted in Section 2.1 on the EPP indicator, to evaluate sensitivity of OVE regarding preparedness and capacity of the health system. Results suggest that OVE could increase to about 60% for $R_e = 2$ if the outbreak is readily detected but could sensibly decrease, even for values of R_e as low as 1.4, 1.6 if hospitalization and number of Ebola beds are lower than assumed. In baseline ring vaccination, we assume hospitalization at 89% and 20 Ebola beds. In Fig. S11 we evaluate sensitivity of OVE regarding impact of ring vaccination. Results show that OVE increases substantially with vaccine coverage at 80% or higher (OVE≈ 60% if $R_e = 2$ with vaccine coverage at 80%). Moreover, OVE drastically increases by adding a spatial component to baseline ring vaccination (OVE close to 90% if $R_e = 2.2$ by adding a spatial ring of 0.5 km, corresponding to targeting the village of the index case). In Fig. S12 we evaluate sensitivity of OVE regarding impact of ring vaccination regarding EVD transmissibility . Results show that OVE increases substantially if

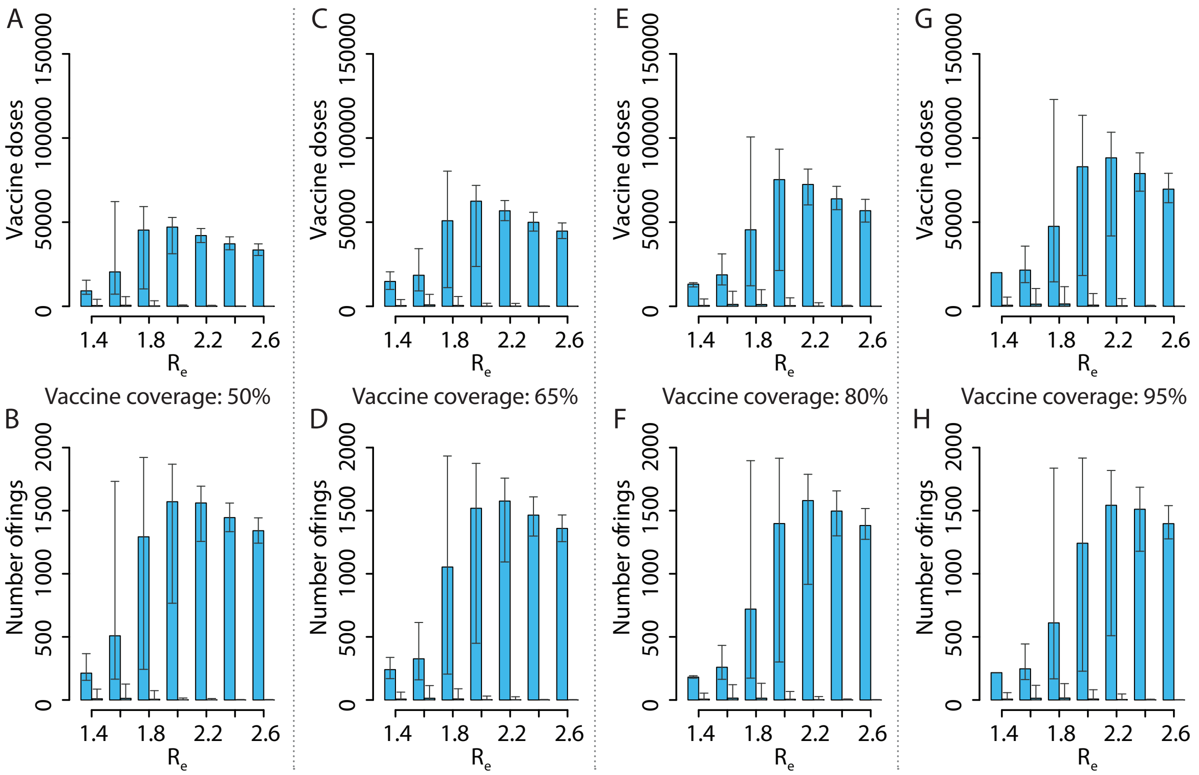

Figure S13: **A** Mean number of vaccine doses (bars) and 95%CI (lines) as a function of R_e by assuming a vaccine coverage of 50%. Both contained (blue bars and lines) and uncontained (green bars and lines) outbreaks are considered. **B** As **A** but for the number of rings. **C** and **D** As **A** and **B** but for vaccine coverage 65% (baseline value). **E** and **F** As **A** and **B**but for vaccine coverage 80%.**G** and **H** As **A** and **B** but but for vaccine coverage 95%.

the time from symptom onset to hospitalization is reduced to 2-3 days on average (OVE close to 100% for $R_e = 2.2$ if the time from symptom onset to hospitalization is reduced to 2 days on average) and by reducing human mobility.

2.4 Vaccine doses and number of rings

Number of vaccine doses and number of rings required to contain an EVD outbreak with baseline ring vaccination have been already discussed in the main text (Fig. 1). If the outbreak is not contained, and $R_e = 1.6$, we found that the number of vaccine doses might be about 20,000 on average, the number of rings might be about 500 on average, with only about 100 rings in the first four months of the outbreak. As shown also in the main text, these numbers drastically decrease in case of contained outbreak. The number of vaccine doses and rings is not sensitive to most of the conducted sensitivity analyses (time required to detect the outbreak, percentage

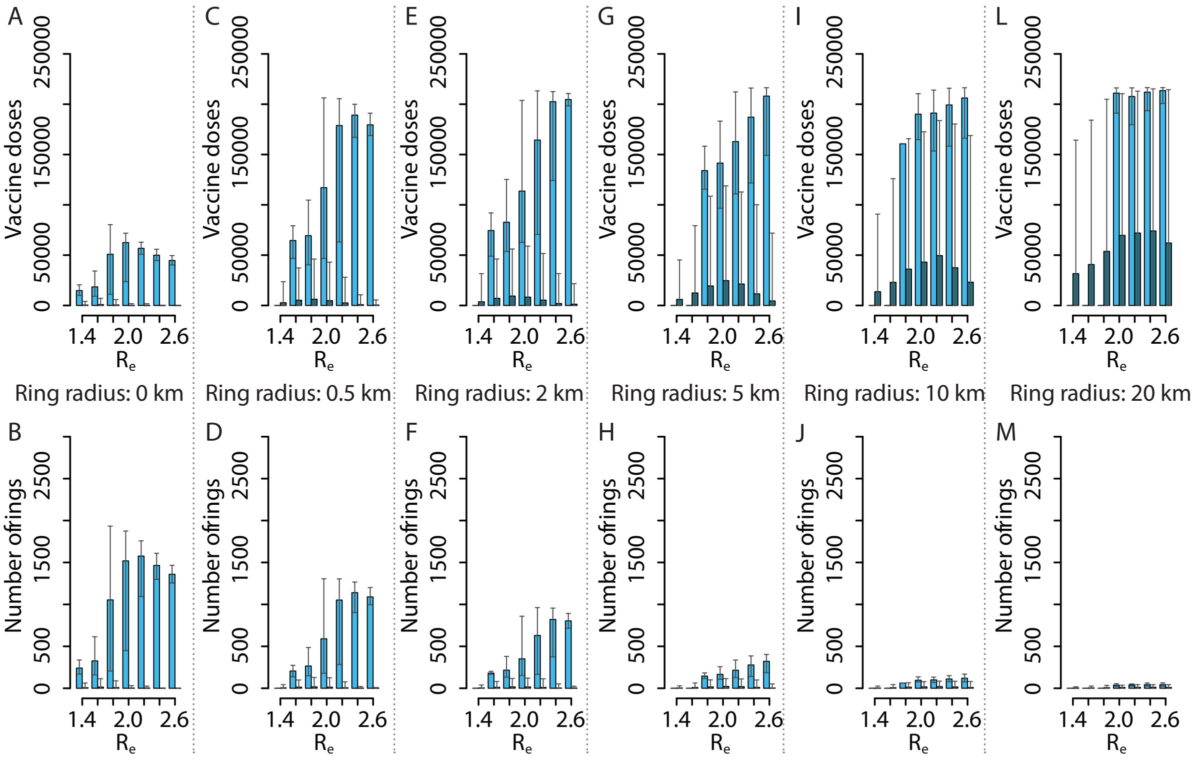

Figure S14: **A** Mean number of vaccine doses (bars) and 95%CI (lines) as a function of R_e by assuming no spatial ring vaccination (ring radius: 0 km, baseline value). Both contained (blue bars and lines) and uncontained (green bars and lines) outbreaks are considered. **B** As **A** but for the number of rings. **C** and **D** As **A** and **B** but considering a spatial ring of radius 0.5 km. **E** and **F** As **A** and **B** but considering a spatial ring of radius 2 km.**G** and **H** As **A** and **B** but considering a spatial ring of radius 5 km.**I** and **J** As **A** and **B** but considering a spatial ring of radius 10 km. **L** and **M** As **A** and **B** but considering a spatial ring of radius 20 km.

of isolated cases, number of available Ebola beds, time for the vaccine to develop protective immunity, human mobility, heterogeneity in transmission, possible impact of additional control measures, e.g. contact tracing). As shown in Fig. S13, the number of vaccine doses and rings does not increase substantially also by assuming vaccine coverage much larger than 65%. Indeed, if vaccine coverage is close to 95%, the number of vaccine doses increases substantially (of about 50%) only for $R_e > 2$ in case of uncontained outbreaks. Of course, the number of vaccine doses might increase dramatically by considering a spatial ring, in case of both contained or uncontained outbreaks. As shown in Fig. S14, the number of vaccine doses might increase of about 300-400% if the ring radius is equal or greater than 2 km. These results suggest that considering a spatial component of the vaccination ring might be difficult to implement in practice in countries with poor resources. However, it is worth noticing that considering a

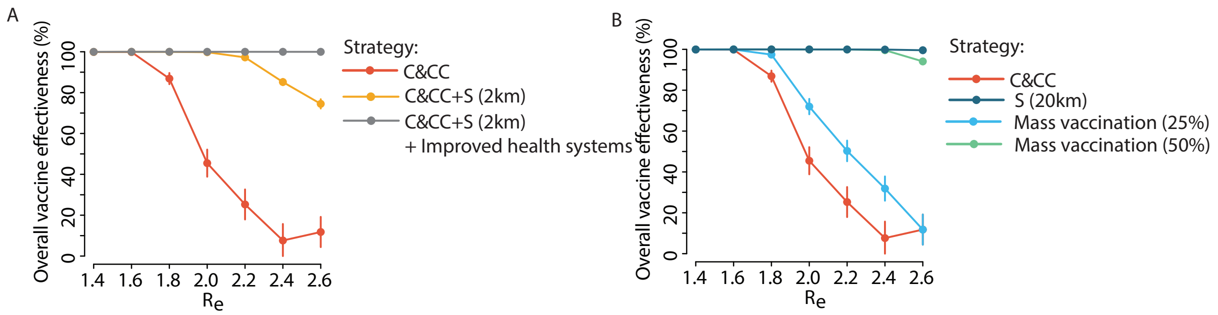

Figure S15: **A** Estimated OVE (± 2 SE) one year after the emergence of the first case (with respect to worst case scenario) as a function of R_e using three ring-defining strategies: contacts and contacts of contacts (C&CC) of index cases; C&CC with a spatial ring of radius 2km around index cases (C&CC+S); C&CC+S (2km) plus "improved health systems", which includes reducing time to isolation of cases (2-3 days from onset to hospitalisation), increasing ring vaccination coverage to 90% of eligible individuals, and reducing public access to infected areas. **B** As A but considering mass vaccination (coverage 25% and 50%) and a spatial ring (S) of 20km around index cases. Each estimate is based on 1,000 simulated outbreaks.

spatial ring of about 0.5 km (roughly corresponding to targeting individuals in the same village of index cases) might be feasible and increase substantially the EPP (results discussed in the main text).

2.5 Outbreak containment: C&CC+S and "improved health systems"

In the main text we have analysed baseline ring vaccination in terms of EPP. All parameter values, with the exception of the number of cases required to detect the outbreak, regulation the impact of interventions are taken from [4, 2]. In particular we assume vaccine coverage at 65%, 4 days on average from symptom onset to hospitalization and eligible population defined as contacts and contacts of contacts of index cases (no spatial ring). Here we analyse the effects of interventions where we assume that vaccine coverage increases to 90%, the time spent in the community by EVD cases is reduced to 2.5 days on average, a spatial component is added to the ring definition (with ring of radius 2 km), and human mobility is reduced (we assumed $b = 3$, resulting in an average distance of about 3.5 km). As shown in the main text, these interventions might lead to contain an EVD outbreak, with OVE close to 100% (Fig. S15A), with R_e as high as 2.6 . As for comparison, OVE for strategies considering spatial rings (20 km) and mass vaccination are shown in Fig. S15B.

References

[1] The DHS Program (2007). Demographic and Health Survey 2007. URL http://dhsprogram.com/Publications/Publication-Search.cfm?ctry_id=22&

country=Liberia.

[2] Henao-Restrepo AM, Longini IM, Egger M, Dean NE, Edmunds WJ, et al. (2015) Efficacy and effectiveness of an rVSV-vectored vaccine expressing Ebola surface glycoprotein: interim results from the Guinea ring vaccination cluster-randomised trial. The Lancet 386: 857–866.

[3] Richards P, Amara J, Ferme MC, Kamara P, Mokuwa E, et al. (2015) Social pathways for Ebola virus disease in rural Sierra Leone, and some implications for containment. PLoS Negl Trop Dis 9(4): e0003567.

[4] Ajelli M, Parlamento S, Bome D, Kebbi A, Atzori A, et al. (2015) The 2014 Ebola virus disease outbreak in Pujehun, Sierra Leone: epidemiology and impact of interventions. BMC Medicine 13:281.

[5] Faye O, Boëlle P, Heleze E, Faye O, Loucoubar C, et al. (2015) Chains of transmission and control of Ebola virus disease in Conakry, Guinea, in 2014: an observational study. Lancet Infect Dis 15: 320-6.

[6] Merler S, Ajelli M, Fumanelli L, Gomes MF, y Piontti AP, et al. (2015) Spatiotemporal spread of the 2014 outbreak of Ebola virus disease in Liberia and the effectiveness of non-pharmaceutical interventions: a computational modelling analysis. The Lancet Infectious Diseases 15: 204–211.

[7] WHO Ebola Response Team (2014) Ebola Virus Disease in West Africa — The First 9 Months of the Epidemic and Forward Projections. N Eng J Med 371: 1481–1495.

[8] Nishiura H, Chowell G (2014) Early transmission dynamics of Ebola virus disease (EVD), West Africa, March to August 2014. Euro Surveill 19: 36.

[9] Althaus CL (2014) Estimating the Reproduction Number of Ebola Virus (EBOV) During the 2014 Outbreak in West Africa. PLOS Currents Outbreaks .

[10] Chowell G, Nishiura H (2014) Transmission dynamics and control of Ebola virus disease (EVD): a review. BMC medicine 12: 196.

[11] Althaus CL (2015) Ebola superspreading. Lancet Infect Dis 15: 507–508.

[12] Ferguson NM, Cummings DA, Cauchemez S, Fraser C, Riley S, et al. (2005) Strategies for containing an emerging influenza pandemic in Southeast Asia. Nature 437: 209–214.

[13] Ferguson NM, Cummings DAT, Fraser C, Cajka JC, Cooley PC, et al. (2006) Strategies for mitigating an influenza pandemic. Nature 442: 448–452.

[14] Merler S, Ajelli M (2010) The role of population heterogeneity and human mobility in the spread of pandemic influenza. Proc R Soc B 277: 557–565.

[15] Merler S, Ajelli M, Pugliese A, Ferguson NM (2011) Determinants of the Spatiotemporal Dynamics of the 2009 H1N1 Pandemic in Europe: Implications for Real-Time Modelling. PLoS Computational Biology 7: e1002205.

[16] Wallinga J, Lipsitch M (2007) How generation intervals shape the relationship between growth rates and reproductive numbers. Proc R Soc B 274: 599–604.

[17] Ajelli M, Merler S (2008) The impact of the unstructured contacts component in influenza pandemic modeling. PLOS ONE 3: e1519.

[18] Chowell G, Hengartner NW, Castillo-Chavez C, Fenimore PW, Hyman JM (2004) The basic reproductive number of Ebola and the effects of public health measures: the cases of Congo and Uganda. J Theor Biol 229: 119–126.

[19] Ajelli M, Merler S (2012) Transmission potential and design of adequate control measures for Marburg hemorrhagic fever. PloS one 7: e50948.

[20] Kiskowski M, Chowell G (2015) Modeling household and community transmission of Ebola virus disease: epidemic growth, spatial dynamics and insights for epidemic control. Virulence : 1–11.

[21] Merler S, Ajelli M, Fumanelli L, Vespignani A (2013) Containing the accidental laboratory escape of potential pandemic influenza viruses. {BMC} Medicine 11: 252.

[22] Mossong J, Hens N, Jit M, Beutels P, Auranen K, et al. (2008) Social contacts and mixing patterns relevant to the spread of infectious diseases. PLoS Med 5: e74.

[23] WHO Global Alert and Response (2014). Situation reports: Ebola response roadmap. URL `http://www.who.int/csr/disease/ebola/situation-reports/en/`.

[24] Lloyd-Smith JO, Schreiber SJ, Kopp PE, Getz WM (2005) Superspreading and the effect of individual variation on disease emergence. Nature 438: 355–359.

[25] Burke J, Declerq R, Ghysebrechts G, Pattyn S, Piot P, et al. (1978) Ebola hemorrhagic-fever in zaire, 1976-report of an international-commission. Bulletin of the World Health Organization 56: 271–293.

[26] Khan AS, Tshioko FK, Heymann DL, Le Guenno B, Nabeth P, et al. (1999) The reemergence of ebola hemorrhagic fever, democratic republic of the congo, 1995. Journal of Infectious Diseases 179: S76–S86.
